# p16-dependent increase of PD-L1 stability regulates immunosurveillance of senescent cells

**DOI:** 10.1038/s41556-024-01465-0

**Published:** 2024-08-05

**Authors:** Julia Majewska, Amit Agrawal, Avi Mayo, Lior Roitman, Rishita Chatterjee, Jarmila Sekeresova Kralova, Tomer Landsberger, Yonatan Katzenelenbogen, Tomer Meir-Salame, Efrat Hagai, Ilanit Sopher, Juan-Felipe Perez-Correa, Wolfgang Wagner, Avi Maimon, Ido Amit, Uri Alon, Valery Krizhanovsky

**Affiliations:** 1 Department of Molecular Cell Biology, Weizmann Institute of Science, Rehovot, Israel; 2 Department of Immunology and Regenerative Biology, Weizmann Institute of Science, Rehovot, Israel; 3 Department of Systems Immunology, Weizmann Institute of Science, Rehovot, Israel; 4 Department of Biological Services, Weizmann Institute of Science, Rehovot, Israel; 5 Institute for Stem Cell Biology, RWTH Aachen University Medical School, Aachen, Germany; 6 Helmholtz Institute for Biomedical Engineering, RWTH Aachen University Medical School, Aachen, Germany

**Keywords:** Senescence, Immune evasion

## Abstract

The accumulation of senescent cells promotes ageing and age-related diseases, but molecular mechanisms that senescent cells use to evade immune clearance and accumulate in tissues remain to be elucidated. Here we report that p16-positive senescent cells upregulate the immune checkpoint protein programmed death-ligand 1 (PD-L1) to accumulate in ageing and chronic inflammation. We show that p16-mediated inhibition of cell cycle kinases CDK4/6 induces PD-L1 stability in senescent cells via downregulation of its ubiquitin-dependent degradation. p16-expressing senescent alveolar macrophages elevate PD-L1 to promote an immunosuppressive environment that can contribute to an increased burden of senescent cells. Treatment with activating anti-PD-L1 antibodies engaging Fcγ receptors on effector cells leads to the elimination of PD-L1 and p16-positive cells. Our study uncovers a molecular mechanism of p16-dependent regulation of PD-L1 protein stability in senescent cells and reveals the potential of targeting PD-L1 to improve immunosurveillance of senescent cells and ameliorate senescence-associated inflammation.

## Abstract (web summary)

Majewska et al. show that p16-expressing senescent cells enhance the stability of the immune checkpoint PD-L1 by downregulating its proteasome-mediated ubiquitin-dependent degradation, leading to their accumulation in ageing and chronic inflammation.

## Main

Senescent cells accumulate in tissues with age, disrupt tissue homeostasis, promote ageing and limit lifespan^1^. Conversely, the elimination of senescent cells substantially delays the onset of age-related phenotypes, including age-associated inflammation (inflammageing), and extends the lifespan in animal models^2,3^. However, the therapeutic potential of senolytic drugs that could selectively clear senescent cells is hampered due to their insufficient specificity and toxicity^4^. Understanding the molecular basis underlying the impaired immunosurveillance of senescent cells, and thus allowing their presence in ageing tissues could provide strategies regulating senescent cell turnover and ameliorating senescence-associated phenotypes.

The age-dependent decline of immune-mediated clearance of senescent cells depends on senescent cell-autonomous mechanisms that inhibit their removal^5,6^, and functional impairment of the aged immune system (immunosenescence)^7,8^. Still, the intrinsic properties of senescent cells limiting their immunosurveillance and mechanisms explaining the immunosenescence-associated increased senescent cell burden in ageing and diseased tissues remain to be elucidated.

In this Article, we used mass cytometry to study senescent cell accumulation in ageing and chronically inflamed lungs in mice. We found that p16-positive senescent cells upregulate protein programmed death-ligand 1 (PD-L1) to evade immunosurveillance and accumulate in ageing and chronic inflammation. Our study reveals the molecular mechanism that regulates PD-L1 stability in senescent cells. p16-mediated inhibition of cyclin-dependent kinases (CDK)4/6 downregulates ubiquitin-dependent proteasomal degradation of PD-L1 leading to its accumulation in senescent cells. Moreover, sequencing of p16-expressing alveolar macrophages (AMs) in chronic lung inflammation has revealed a transcriptional immunosuppressive signature coupled to p16 expression. We show that activating PD-L1 antibody engaging Fcγ receptors on effector cells enhances immune-mediated clearance of senescent cells in vivo. Consistent with this, the administration of these activating PD-L1 antibodies to naturally ageing mice and mice with chronically inflamed lungs reduces p16 and PD-L1 double-positive senescent cells, and ameliorates senescence-associated inflammation.

## Results

### p16-positive senescent cells upregulate immune checkpoint PD-L1 in ageing and chronic inflammation

**Fig. 1:**
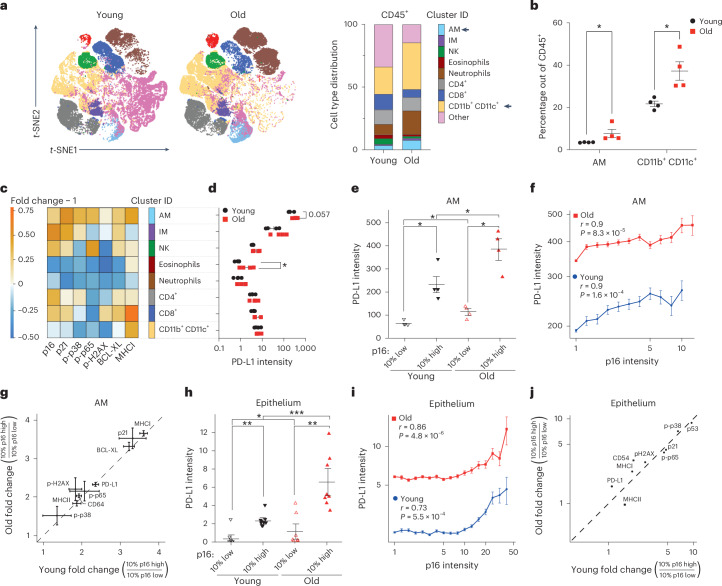
PD-L1 upregulation correlates with p16 expression in ageing. **a**, Representative *t*-SNE plots of immune cell (CD45^+^) populations identified in 50,000 cells from all lung samples of young and old mice (left) and their immune cell type distribution (right). Significantly changed populations are marked with an arrow (right). **b**, The frequency of indicated immune cell types in young and old mice. **c**, The heatmap of the change in the enrichment of senescence-related proteins in old/young mice for indicated immune cell types. **d**, Mean expression of PD-L1 in the identified immune cell populations. **e**, Mean expression of PD-L1 in 10% highest and 10% lowest p16-expressing cells within the AM population in young and old mice. **f**, Spearman correlation between p16 and PD-L1 protein expression in AMs in young and old mice. The AM cells were binned into 12 bins in old mice with a median of 740 cells per bin, and 11 bins in young mice with a median of 350 cells per bin. **g**, Scatter plot showing fold change in expression of senescence-related markers between p16 high and p16 low expressing cells in the AMs in the young (*n* = 4) and old (*n* = 4) mice. The diagonal line marks an equal fold change between young and old. **h**, Mean expression of PD-L1 between 10% highest and 10% lowest p16-expressing cells within lung epithelium in young and old mice. **i**, Spearman correlation between p16 and PD-L1 expression in the lung epithelium in young and old mice. The epithelium cells in young and old mice were binned into 18 bins with a median of 14,000 cells per bin. **j**, Scatter plot showing fold change in expression of senescence-related markers between p16 high and p16 low expressing cells in the epithelium in young (*n* = 8) and old (*n* = 8) mice. In **f** and **i**, Spearman correlation coefficient (*r*) and associated *P* value (*P*) were used for statistical analysis. Single cells were ranked by p16 expression level in bins from low to high. For each bin, the mean expression level of PD-L1 is shown. Two-sided (**b** and **d**) or one-sided (**e**–**j**) Mann–Whitney *U* test was used for statistical analysis. Error bars, mean ± s.e.m. *P < 0.05, **P < 0.01, ***P < 0.001. Young (*n* = 4) and old (*n* = 4) mice were used in **b**–**g**, and young (*n* = 8) and old (*n* = 8) mice were used in **h**–**j**. Experiments were repeated three times independently with similar results. Source numerical data are available in Source data. Source data

**Extended Data Fig. 1:**
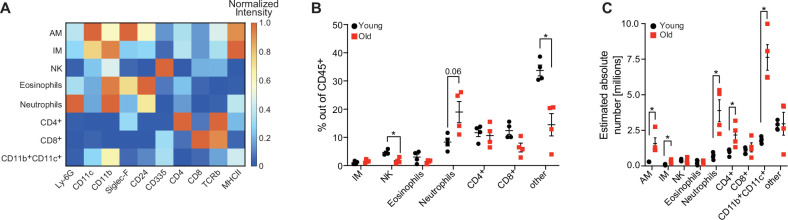
PD-L1 upregulation correlates with p16 expression in aging. **(A)** The heatmap of normalized lineage markers expression for identified immune cell types. The relative frequency **(B)** and absolute number **(C)** of indicated immune cell types in young and old mice. Young (n = 4), old (n = 4). Two-sided Whitney-Mann U test was used for statistical analysis. Error bars, mean ± SEM. *p < 0.05, **p < 0.01, ***p < 0.001. Experiments were repeated three times independently with similar results. Source numerical data are available in source data. Source data

Immunosenescence has a causal role in driving systemic ageing, including an increased burden of senescent cells^7^. To unravel cell-autonomous mechanisms leading to impaired immunosurveillance of senescent cells, we investigated functional phenotypes of immune cells undergoing senescence with age. We performed a mass cytometry analysis on lung immune cells from young 2-month-old and aged 24-month-old C57BL/6 wild-type mice. The two-dimensional reduction ‘viSNE’ (Vi-distributed stochastic neighbor embedding) and FlowSOM-based clustering of the CD45^+^ immune cells distinguished AMs and interstitial macrophages (IMs), natural killer (NK) cells, eosinophils, neutrophils, CD4^+^ and cytotoxic CD8^+^ T cells, and CD11b^+^CD11c^+^ myeloid cells (Fig. 1a and Extended Data Fig. 1a). In aged lungs, the frequencies of AMs and CD11b^+^CD11c^+^ myeloid cells were significantly increased, both the relative and absolute cell numbers (Fig. 1b and Extended Data Fig. 1b,c). We then analysed each of the identified immune cell types for the expression of senescence-related proteins. Remarkably, AMs were particularly enriched for senescence (p16 and p21), pro-inflammatory (p-p38 and p-p65) and immunoregulatory (major histocompatibility complex I; MHCI) markers in aged mice compared with young counterparts (Fig. 1c). Strikingly, AM also showed the highest expression of immune checkpoint protein PD-L1 comparing to all other immune subsets (Fig. 1d). Of note, AMs showed a significantly increased level of PD-L1 as a function of p16 expression, which was further pronounced in ageing (Fig. 1e,f). Single-cell analysis of AMs revealed a positive correlation between p16 and PD-L1 (Spearman’s rank correlation coefficient of the young (0.9) and old (0.9) mice; Fig. 1f). We then wanted to understand if p16 expression is coupled with the other proteins related to the senescent phenotype. We analysed fold change of expression of senescence-related proteins as a function of p16 level and found that cyclin-dependent kinase inhibitor p21 (Cdkn1a), anti-apoptotic protein B cell lymphoma extra large (BCL-XL) and MHCI showed the most striking correlation with p16, irrespective of the age of mice (Fig. 1g). Increased expression of p21 and BCL-XL is known to underlie senescent cells’ resistance to apoptosis and can contribute to their accumulation in tissues^9,10^. To understand if the observed phenomena is not limited to AMs, we analysed lung epithelium, which is known to accumulate senescent cells in ageing^11,12^. Similarly to AMs, epithelial cells showed significant upregulation of PD-L1 expression as a function of p16 expression in both young and old mice, but again with higher levels in old mice (Spearman’s rank correlation coefficient of young (0.73) and old (0.86) mice; Fig. 1h,i). We found that expression of core proteins of the senescence machinery (p21 and p53), followed by proteins involved in inflammatory response (p-p65 and p-p38) showed the strongest correlation with p16 level, regardless of age (Fig. 1j). These results indicate that the expression of p16 is correlated with PD-L1 expression in senescent cells, with higher levels of expression of both proteins in older mice (Fig. 1e,f,h,i). We hypothesized that macrophages with senescent phenotype might contribute to inflammation, but also induce a compensatory immunosuppressive response to prevent excessive tissue damage. This is in agreement with a recently reported subset of p16-expressing AMs with senescence-like properties in ageing lungs, which suppress cytotoxic T cell responses^13^. Thus, in ageing, increased expression of p16 associated with PD-L1-mediated immunosuppression might lead to compromised immunosurveillance and consequently increased senescent cell burden.

**Fig. 2:**
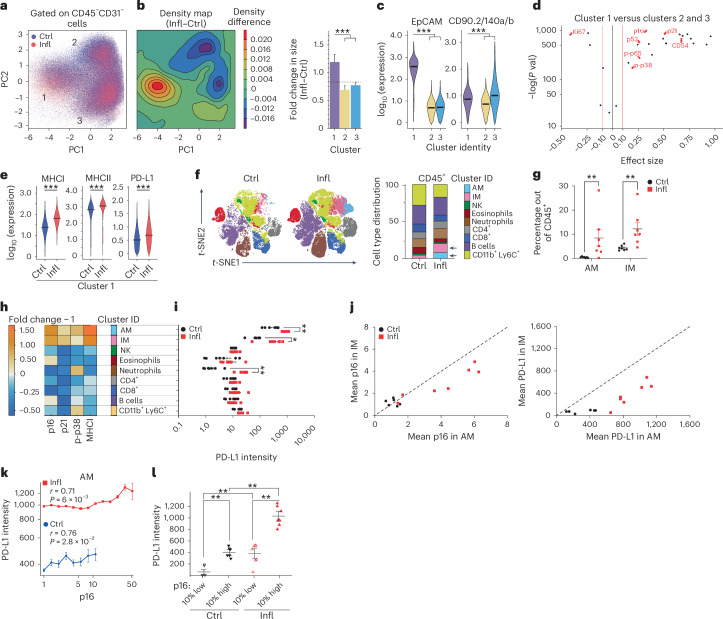
PD-L1 upregulation correlates with p16 expression in chronic inflammation. **a**, Principal component analysis of CD45^−^CD31^−^ cells from control (PBS, Ctrl) and chronically inflamed (LPS, Infl) lungs identified three clusters (1, 2 and 3) based on their principal component (PC) values. **b**, Cell density map for each cluster shown as a difference in Kernel density distribution between Ctrl and Infl conditions. The quantification of the fold change of cell frequency for each cluster is shown (right). Error bars were estimated by bootstrapping. A total of 1 × 10^4^ cells were sampled from each cluster with sampling repeated 1 × 10^4^ times. **c**, The expression intensity distribution of epithelial marker (EpCAM) and fibroblast markers (CD90.2, CD140a and CD140b) for identified clusters. Statistical significance was calculated by the Kruskal–Wallis test. **d**, Volcano plot displaying enrichment of senescence-related proteins and depletion of the proliferation marker Ki-67 within cluster 1 in comparison with clusters 2 and 3. The black line marks the effect size equal to 0. The left and right red lines mark the −0.1 and 0.1 effect size thresholds, respectively. **e**, The expression intensity distribution of the indicated proteins between Ctrl and Infl mice within cluster 1. **f**, Representative *t*-SNE plots of immune cell (CD45^+^) populations identified in 50,000 cells from all lung samples of Ctrl and Infl mice (left). The immune cell types distribution in Ctrl and Infl mice are shown, and significantly changed populations of AMs and IMs marked with an arrow (right). **g**, Frequency of AM and IM in the lungs of Ctrl and Infl mice. **h**, The change in the enrichment of senescence-related proteins in Infl/Ctrl mice for indicated immune cell types. **i**, Mean expression of PD-L1 between identified immune cell populations. **j**, Mean p16 (left) and PD-L1 (right) expression between AMs and IMs. **k**, AMs were binned into 8 bins in Ctrl and 13 bins in Infl with a median of 80 and 1,900 cells per bin, respectively. Spearman correlation coefficient (*r*) between p16 and PD-L1 expression in AMs of Ctrl and Infl mice and associated *P* value (*P*). Single cells were ranked by p16 expression level in bins from low to high. For each bin, the mean expression level of PD-L1 is shown. **l**, Mean PD-L1 expression in the 10% highest and 10% lowest p16-expressing cells within AM in the lungs of Ctrl and Infl mice. One-sided (**e** and **k**–**l**) and two-sided (**g** and **i**) Mann–Whitney *U* tests was used for statistical analysis unless otherwise noted. Error bars, mean ± s.e.m. *P < 0.05, **P < 0.01, ***P < 0.001. In **a**–**l**, Ctrl (*n* = 5–7) and Infl (*n* = 5–7) lungs were used. Experiments were repeated three times independently with similar results. Source numerical data are available in Source data. Source data

**Extended Data Fig. 2:**
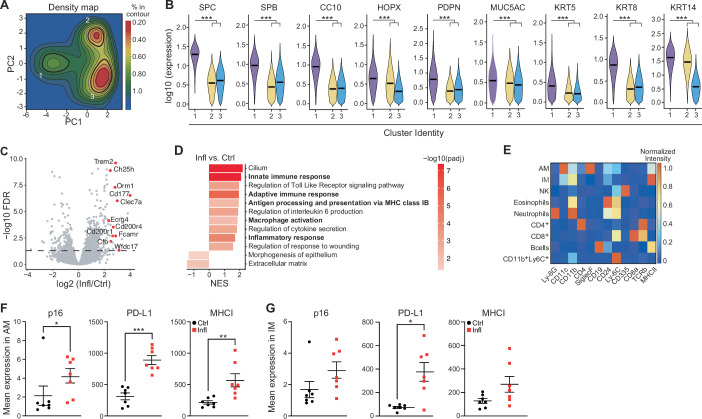
PD-L1 upregulation correlates with p16 expression in chronic inflammation. **(A)** Cell density map for each cluster as a function of Kernel density distribution in Ctrl and Infl condition. **(B)** Violin plot displaying the expression intensity distribution of phenotypic markers for identified clusters. Statistical significance was calculated by the Kruskal-Wallis test. **(C-D)** RNA-seq of lung epithelial cells derived from lungs of Ctrl (n = 5) and Infl (n = 3) mice **(C)** Volcano plot of differentially expressed genes (DEGs) between Ctrl and Infl mice (n = 5). DEGs were identified by Padj < 0.05 and |Fold change | > 1.25. Immune response-related genes are labeled in red. **(D)** Gene Ontology (GO) enrichment analysis of DEGs identified in (C). GO terms are ordered by Normalized Enrichment Score (NES). A positive NES value indicates enrichment in Infl condition, negative NES value indicates enrichment in the Ctrl phenotype. The color of the bar denotes -log10 (p-value). **(E)** Heatmap of normalized lineage marker expression for indicated immune cell types. **(F-G)** Comparison of mean expression of p16, PD-L1, and MHCI markers within **(F)** AM and **(G)** IM in Ctrl and Infl mice (n = 7). (F-G) Two-sided Mann-Whitney test was used for statistical analysis. Error bars, mean ± SEM. *p < 0.05, **p < 0.01, ***p < 0.001, (A-G) control (n = 5−7), Infl (n = 5-7). A-B and E-G experiments were repeated three times independently with similar results. Source numerical data are available in source data. Source data

Previously, we have reported that accumulating senescent lung epithelial cells promote chronic lung inflammation^14^. We therefore asked if p16-positive senescent cells upregulate PD-L1 in chronically inflamed lungs, which might explain their compromised immune clearance. To address this question, we used a mouse model of lipopolysaccharide (LPS)-induced chronic lung inflammation, which resembles the pathology of chronic obstructive pulmonary disease^15^. To comprehensively characterize senescence in the epithelium, we took an unbiased mathematical approach to study cellular and phenotypic diversity of p16-positive senescent cells within the non-immune, non-endothelial cell compartment (CD45^−^CD31^−^) in chronic lung inflammation by mass cytometry. Principal component analysis of the data identified three distinct clusters (designated as clusters 1, 2 and 3) within this cell population derived from inflamed (LPS) and control (phosphate-buffered saline (PBS) lungs (Fig. 2a and Extended Data Fig. 2a). Density of cells within cluster 1, but not the other two clusters, significantly increased upon chronic inflammation (Fig. 2b). The cells within cluster 1 were significantly enriched in the expression of epithelial marker EpCam and other canonical markers of lung epithelium subsets (SPC/SPB—alveolar cell type II, HOPX/PDPN—alveolar cell type I, CC10—club cells, Muc5AC—goblet cells, KRT5/KRT14—basal cells and KRT8—alveolar progenitor cells; Fig. 2c, left, and Extended Data Fig. 2b), indicating their epithelial identity. The cells in cluster 2 showed expression of the basal progenitor marker KRT14 (Extended Data Fig. 2b), and cluster 3 was enriched in fibroblast markers (CD90.2, CD140a and CD140b) (Fig. 2c, right). Analysis of senescence markers in cells of cluster 1 showed enrichment for proteins of cell cycle arrest (p16, p21 and p53), and depletion of the proliferation marker Ki-67 (Fig. 2d). The cells in cluster 1 were also enriched for molecules involved in the pro-inflammatory pathways (p-p38, p-p65 and CD54), which can promote chronic inflammation (Fig. 2d). Remarkably, the cells in cluster 1 also upregulated molecules associated with antigen presentation (MHCI and MHCII) and the immune checkpoint molecule PD-L1 (Fig. 2e), similar to what we observed in the lungs of old mice. These results suggest that p16-positive senescent cells with enhanced MHC machinery express PD-L1, thus providing an intrinsic mechanism for their escape from immunosurveillance, and explaining their role in chronic inflammation by dysregulating immune responses.

Our next aim was to further understand how epithelial cells mediate the dysregulation of immune responses upon chronic damage. To address this, we performed RNA sequencing (RNA-seq) of purified lung epithelial cells from LPS-treated and control mice. Gene Ontology enrichment analysis of genes upregulated in the lung epithelium of mice with chronic inflammation indicated a marked increase in the expression of genes involved in a pro-inflammatory response, antigen processing and presentation (Extended Data Fig. 2c,d). Furthermore, we observed an increase in gene signatures related to the activation of adaptive and innate immune responses, suggesting that the epithelial compartment enriched with senescence enhances the activation of macrophages during chronic inflammation.

We further hypothesized that chronic inflammation might promote immune cells to undergo senescence, which in turn comprises the immune cell function and results in the higher burden of damaged or senescent cells. We evaluated immune cell populations using a senescence and immune–cell-centric antibody panel. viSNE analysis and FlowSOM-based clustering of CD45^+^ immune cells distinguished AM, IM, NK cells, eosinophils, neutrophils, CD4^+^ and CD8^+^ T cells, B cells, and CD11b^+^Ly6C^+^ myeloid cells (Fig. 2f and Extended Data Fig. 2e). In chronically inflamed lungs, we observed a significant increase in AMs and IMs (Fig. 2g). We analysed all the identified immune cell populations for the expression of senescence-related proteins. Only AM and IM were enriched for the expression of senescence (p16 and p21), pro-inflammatory (p-p38) and immunoregulatory (MHCI) markers in chronically inflamed compared with control mice (Fig. 2h and Extended Data Fig. 2f,g). The macrophages also showed the highest expression of PD-L1 protein compared with all other immune subsets, both in steady state and during inflammation (Fig. 2i). However, the mean expression levels of p16 and PD-L1 were higher in AMs than in IMs (Fig. 2j). Of note, the expression of p16 in AMs was significantly correlated with PD-L1 levels, consistent with our observation of age-related changes in AMs (Spearman’s rank correlation coefficient of LPS (0.71) and PBS (0.76); Fig. 2k,l). Together, these results show that chronic inflammation triggered the accumulation of macrophages with a senescent phenotype and induced the expression of PD-L1 in these cells.

AMs and the epithelial lining of the respiratory tract are the first lines of cellular and physical defence against environmental hazards such as cigarette smoke, allergens and other air-born pollutants^16,17^. Pulmonary epithelium also interacts with immune cells to maintain homeostasis while facilitating immune response when necessary^16^. Senescence of lung epithelium promotes chronic lung inflammation^14^ and upregulation of PD-L1 in both senescent epithelial cells, and macrophages with senescent phenotype might be an intrinsic mechanism to evade immune clearance and promote persistent senescence in the tissue. We suggest that the upregulation of immune checkpoint PD-L1 on damage-induced senescent cells minimizes autoreactive immune responses in chronic inflammation to preserve organ integrity and function.

### p16-mediated inhibition of CDK4/6 links decreased ubiquitination to the upregulation of PD-L1 stability in senescence

**Fig. 3:**
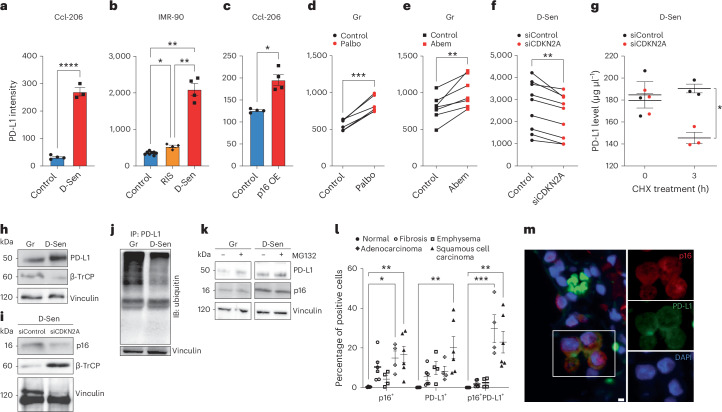
p16 increases the stability of PD-L1 protein in senescent cells. **a**–**c**, Flow cytometry analysis of PD-L1 expression in DNA damage-induced senescence (D-Sen) (**a** and **b**), RIS (**b**) and in cells with p16 overexpression (p16 OE) (**c**) compared with control cells. Primary mouse lung fibroblasts (CCL-206) (**a** and **c**) and primary human lung fibroblasts (IMR-90) (**b**) were used in these experiments (*n* = 3–8). **d**,**e**, PD-L1 protein expression in growing IMR-90 cells treated with CDK4/6 inhibitors Palbociclib (Palbo) (**d**), Abemaciclib (Abem) (**e**) or vehicle (control) (*n* = 6–7). Gr, growing. **f**, PD-L1 protein expression in D-Sen treated with non-targeting small interfering RNA (siControl) or small interfering CDKN2A (siCDKN2A) (*n* = 9). **g**, ELISA-based measurement of PD-L1 protein levels in D-Sen treated with siControl or siCDKN2A and CHX (*n* = 3). **h**–**k**, Immunoblot analysis of whole cell lysates derived from Gr and D-Sen cells (**h**), D-Sen cells treated with siControl and siCDKN2A (**i**), Gr and D-Sen cells treated with MG132 or vehicle (negative control) (**k**) (*n* = 3), and immunoblot (IB) analysis of ubiquitin in immunoprecipitated (IP) PD-L1 protein from Gr and D-Sen cells (**j**). **l**, Quantification of p16^+^, PD-L1^+^ and p16^+^PD-L1^+^ cells in normal lung tissue (*n* = 3) and human lung pathologies: emphysema (*n* = 3), fibrosis (*n* = 3), adenocarcinoma (*n* = 3) and squamous cell carcinoma (*n* = 3). **m**, Representative immunofluorescence image of p16 (red) and PD-L1 (green) staining in emphysema patient. Blue, nuclei stained by DAPI. Scale bar, 10 μm. The image is representative of *n* = 4 emphysema lung specimens. PD-L1 expression in **a**–**f** was quantified by flow cytometry analysis as median fluorescent intensity. Two-tailed unpaired Student’s *t*-test (**a**, **c** and **g**) and two-tailed paired Student’s *t*-test (**d**, **e** and **f**) was used. Error bars, mean ± s.e.m. *P < 0.05, **P < 0.01, ***P < 0.001. One-way ANOVA (**b** and **l**) was also used for statistical analysis; error bars, mean ± s.e.m. **P < 0.01, ****P < 0.0001. In **a**–**k**, experiments were repeated three times independently with similar results. Source numerical data and unprocessed blots are available in Source data. Source data

**Extended Data Fig. 3:**
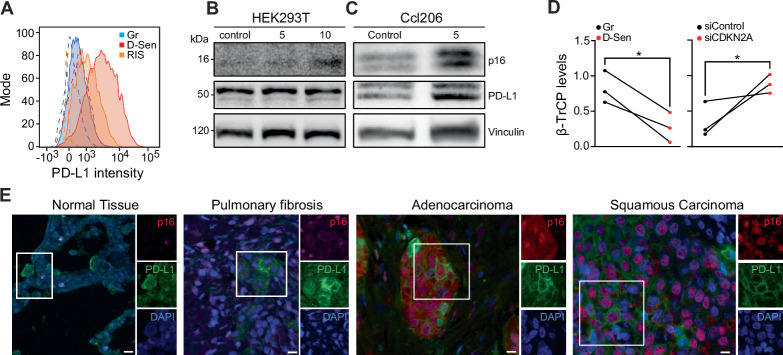
p16 increases the stability of PD-L1 protein in senescent cells. **(A)** Representative histogram showing PD-L1 level in growing (Gr, blue), replicative senescence (RIS, orange), and DNA-damage-induced senescence (D-Sen, red) by flow cytometry. Isotype control for each condition is presented by a dashed line. **(B-C)** Immunoblot (IB) analysis of whole cell lysates derived from (B) HEK293T cells, (C) CCL-206 cells treated with vehicle (Control) or doxycycline (5 μg/ml or 10 μg/ml) to induce p16 overexpression (p16 OE). **(D)** B-TrCP levels from immunoblot analysis of IMR90 cells (D, left) growing (Gr) and D-Sen and (D, right) D-Sen cells treated with siControl and siCDKN2A. Two-tailed Student t-test; Error bars, mean ± SEM. *p < 0.05. **(E)** Representative immunofluorescence images of p16 (red) and PD-L1 (green) staining in normal lung tissue, and lungs in patients with pulmonary fibrosis, lung adenocarcinoma, and squamous cell carcinoma. Blue, nuclei stain by DAPI. Scale bars indicate 20 μm. Images are representative of normal (n = 4), fibrosis (n = 6), adenocarcinoma (n = 4), and squamous cell carcinoma (n = 6) lung specimens. B-D experiments were repeated independently three times with similar results. A-D experiments were repeated three times independently with similar results. Source numerical data and unprocessed blots are available in source data. Source data

To explore the molecular mechanism behind the elevation of PD-L1 in senescent cells, we used a classical model of cellular senescence in primary mouse (Ccl-206) and human (IMR-90) lung fibroblasts induced to senesce by DNA damage^10^. Senescence induction significantly increased surface PD-L1 expression compared with growing control cells (Fig. 3a,b and Extended Data Fig. 3a). Replicative exhaustion, another physiological inducer of senescence, also caused PD-L1 upregulation (Fig. 3b and Extended Data Fig. 3a). p16 overexpression induced elevation of PD-L1 protein levels in mouse, but not in human cells (Fig. 3c and Extended Data Fig. 3b,c). Under in vitro culture conditions, mouse cells accumulate DNA damage faster than human cells, which might explain interspecies differences in p16 overexpression and change in PD-L1 level. Interestingly, cells induced to senesce by overexpression of p16 (and without induced DNA damage), do not express senescence-associated secretory phenotype components despite other hallmarks of senescence^18^. These results suggest that regardless of the species, DNA damage-mediated, senescence-associated inflammation plays a key role in the regulation of PD-L1 expression with p16 further promoting its increased protein levels.

p16 inhibits CDK4/6 to induce proliferative arrest in senescent cells. To test if PD-L1 upregulation is enhanced by p16-mediated inhibition of CDKs, we treated growing primary fibroblasts with highly selective inhibitors of CDK4 and CDK6 (CDK4/6i), abemaciclib or palbociclib, which can be considered functional p16 mimetics^19^. Treatment with CDK4/6i significantly elevated surface PD-L1 expression (Fig. 3d,e). To understand whether the observed increase of PD-L1 protein in senescent cells is mediated by p16-dependent inhibition of CDK4/6, we silenced gene-encoding p16 (Cdkn2a) in senescent cells. We found that this knockdown efficiently reduced PD-L1 protein levels in senescent cells (Fig. 3f). Since inhibition of proteasome-mediated degradation induces stability of many proteins to ensure cellular integrity in response to stress^20^, we hypothesized that this mechanism might be responsible for p16-mediated PD-L1 upregulation. To examine the effect of p16 on the stability of endogenous PD-L1 protein in senescent IMR-90 cells, we used a cycloheximide (CHX) chase assay to block translation and measure the degradation rate of PD-L1 protein. Knockdown of p16 caused a significant acceleration of PD-L1 turnover 3 h post CHX treatment (Fig. 3g), indicating that PD-L1 degradation is p16 dependent. These results demonstrate that p16-mediated inhibition of CDK4/6 promotes PD-L1 protein stability in cellular senescence.

E3 ubiquitin ligases control PD-L1 protein levels through ubiquitin-dependent proteasome-mediated protein degradation^21^. SKP1-CUL1-F-box protein (SCF) E3 ubiquitin ligases constitute the largest family responsible for the turnover of key regulatory proteins of the cell cycle^22^. β-TrCP is a substrate recognition component of SCF complex, also known to mediate PD-L1 ubiquitination for subsequent degradation^21^. We reasoned that in cellular senescence the activity of SCF might be altered, thus affecting PD-L1 ubiquitination. We found that β-TrCP levels in senescent cells are downregulated compared with growing control cells (Fig. 3h and Extended Data Fig. 3d), possibly contributing to elevated PD-L1 levels in senescence (Fig. 3h). Depletion of p16 in senescent cells restored β-TrCP level, possibly explaining the accelerated degradation of PD-L1 in senescent cells with p16 knockdown (Fig. 3g,i and Extended Data Fig. 3d). Given that SCF ubiquitin ligase governs the ubiquitination and degradation of PD-L1 via β-TrCP, we evaluated its ubiquitination levels in senescent cells. Immunoprecipitation of PD-L1 indicated a significantly lower ubiquitination signal in senescent cells compared with growing (Fig. 3j). Treatment with the proteasome inhibitor MG132 showed a trend of PD-L1 protein increase in growing cells but did not affect PD-L1 levels in senescent cells, further indicating a reduced proteasomal degradation of PD-L1 in senescent cells (Fig. 3k). These results indicate that p16 in senescence is linked to the upregulation of PD-L1 stability via decreased proteasome-mediated degradation.

Multiple mechanisms regulate PD-L1 at the transcriptional, post-transcriptional and post-translational levels^23^. The pro-inflammatory secretome of senescent cells can upregulate PD-L1 mRNA expression through JAK/STAT pathway^24^. In cancer cells, cyclin D–CDK4/6-dependent phosphorylation of speckle-type POZ protein (SPOP)/Cullin-3 ubiquitin ligase complex destabilizes PD-L1 via proteasome-mediated degradation, but inhibition of CDK4/6 significantly elevates PD-L1 level^25^. Interestingly, in tumour cells the expression of p16 strongly correlates with PD-L1 levels, highlighting the therapeutic potential of PD1–PD-L1 immune checkpoint therapy^25,26^. Similarly, in lung adenocarcinoma and squamous cell carcinoma patients, we observed a significant accumulation of cells expressing both p16 and PD-L1 (Fig. 3l and Extended Data Fig. 3e). Furthermore, in patients with emphysema, a tissue manifestation of chronic obstructive pulmonary disease and lung fibrosis, we also identified p16, PD-L1 double-positive cells (Fig. 3l,m and Extended Data Fig. 3e). This data suggests that p16-positive cells that co-express PD-L1, which suppress the anti-tumour response, could also contribute to immune suppression in ageing, leading to chronic lung disease. Together, our results link p16-mediated inhibition of CDK4/6 and compromised ubiquitination to the upregulation of PD-L1 stability, which could lead to potential strategies to enhance the immune response against PD-L1 in senescent cells.

### p16-positive AMs show immunosuppressive phenotype

**Fig. 4:**
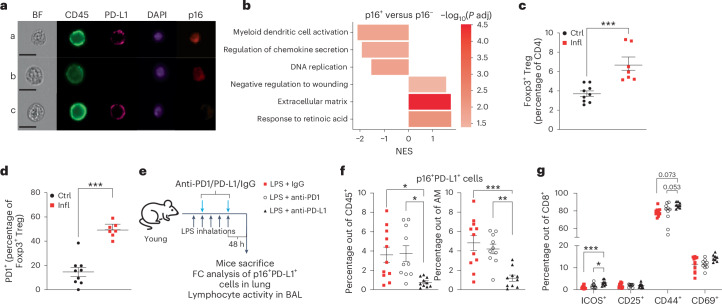
Anti-PD-L1, but not anti-PD1, antibody depletes p16, PD-L1-positive cells in vivo*.* **a**, Imaging flow cytometry analysis of subcellular localization of p16 and PD-L1 staining within the AM population. Representative images of CD45^+^PD-L1^+^p16^+^ (row a), CD45^+^PD-L1^−^p16^+^ (row b) and CD45^+^PD-L1^+^p16^−^ (row c) cells. Bright field (BF); scale bars, 10 μm. Images are representative from three mice repeated independently with similar results. **b**, INs-seq of p16^+^ and p16^−^ AMs. GSEA of p16^+^ and p16^−^ AMs. Control (*n* = 4) and inflamed (Infl; *n* = 4) samples were used. DESeq2 was used to derive gene fold changes for p16^+^ versus p16^−^ macrophages, controlling for treatment (LPS/PBS) as a covariant. NES, normalized enrichment score. **c**,**d**, Flow cytometry analysis of lung with the percentage of Foxp3^+^ Tregs within CD4 population (**c**) and the percentage of CD4 Foxp3^+^ Tregs expressing PD1 (**d**). Ctrl, control (PBS); Infl, inflamed (LPS). **e**, Experimental setup: the mice that were exposed daily to either PBS (Ctrl) or LPS (Infl) inhalations for 5 days received anti-PD1, anti-PD-L1 or matched IgG control as indicated, and the lungs and BAL were analysed 48 h after the last inhalation. FC, flow cytometry. **f**,**g**, Flow cytometry analysis of lung (**f**) or BAL (**g**) from the mice treated as in **e**. **f**, Percentage of p16^+^PD-L1^+^ cells within CD45^+^ or AM cells. **g**, Percentage of CD8^+^ T cells positive for ICOS, CD25, CD44 and CD69. Two-sided Mann–Whitney *U* test (**c** and **d**) was used for statistical analysis. Error bars, mean ± s.e.m., ***P < 0.001. In **f** and **g**, one-way ANOVA was used for statistical analysis. Error bars, mean ± s.e.m., *P < 0.05, **P < 0.01, ***P < 0.001. In **b** and **d**, control (*n* = 5–9) and Infl (*n* = 5–7) samples were used. In **f** and **g**, LPS + IgG (*n* = 11), LPS + anti-PD1 (*n* = 10) and LPS + anti-PD-L1 (*n* = 10) samples were used. In **c**–**g**, experiments were repeated three times independently with similar results. Source numerical data are available in Source data. Source data

We observed that cellular senescence triggers p16-mediated induction of PD-L1 in ageing and chronic inflammation. So far, it is unknown if p16 expression contributes to compromised immune surveillance. Therefore, we decided to investigate the functional significance of p16-positive cells in this process, focusing on AM, as an example, due to their distinct accumulation in chronic lung inflammation (Figs. 2g and 4a). We used a sequencing technology that combines intracellular labelling with transcriptomics, hereafter referred to as INs-seq, to study p16-expressing cells^27^. Gene set enrichment analysis (GSEA) revealed marked upregulation of central genes involved in extracellular matrix remodelling or the response to retinoic acid in p16 high-expressing AMs (Fig. 4b). Remarkably, p16 expression was negatively correlated not only with the DNA replication, but also with immune responses, including dendritic cell activation or chemokine secretion (Fig. 4b). AMs contribute to respiratory tolerance via retinoic acid-mediated induction of Foxp3 expression in naive T cells^28^. In this line, in chronically inflamed lungs, we observed a significant increase of Foxp3^+^ regulatory T cells (Tregs) enriched with marked PD1 expression (Fig. 4c,d). Interestingly, Tregs secrete regulatory cytokines, such as interleukin (IL)-4 and IL-10, which can modulate anti-inflammatory macrophage phenotype suggesting the cross-regulation of the immune response^28^. Together, these results suggest that high-level expression of p16 in AMs induces an immunosuppressive environment with compromised immune responses and might lead to non-resolving inflammation upon chronic damage.

### Anti-PD-L1, but not anti-PD1, antibody treatment depletes p16-, PD-L1-positive cells in vivo

**Extended Data Fig. 4:**
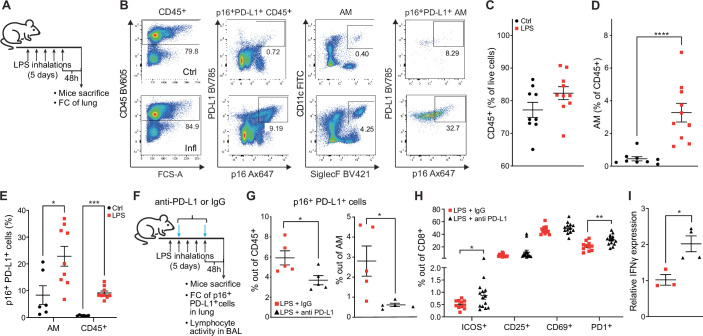
Anti-PD-L1, but not anti-PD-1, antibody treatment depletes p16, PD-L1^−^positive cells *in vivo.* **(A)** Experimental setup: mice were exposed daily to either PBS (Ctrl) or LPS inhalations for 5 days before analysis of mice lungs 48 h after the last inhalation. **(B)** Representative flow cytometry plots showing the gating strategy for p16^+^ PD-L1^+^ cells from immune cells (CD45^+^) and Alveolar Macrophage (AM) population. **(C-E)** Flow cytometry analysis of **(C)** CD45^+^, **(D)** AM, and **(E)** p16^+^PD-L1^+^ cells within CD45^+^ and AM populations in mice treated as described in A. (n = 6-10) **(F)** Experimental setup: mice exposed daily to either PBS (Ctrl) or LPS inhalations for 5 days received anti-PD-L1 or matched IgG control as indicated, and lungs and bronchoalveolar lavage (BAL) were analysed 48 h after the last inhalation. **(G-H)** Flow cytometry analysis of lung **(G)** or BAL **(H)** from mice treated as in scheme F. **(G)** Percentage of p16^+^PD-L1^+^ cells within CD45^+^ or AM. (n = 5) (**H)** Percentage of CD8 T cells positive for ICOS, CD25, CD69, and PD1 (n = 13-14) **(I)** mRNA level of *IFN-γ* cytokine from CD8 T cells in BAL. Values are relative to mice treated with IgG control (n = 3-4). (C-E, G-I) Two-sided Mann-Whitney test was used for statistical analysis. Error bars, mean ± SEM *p < 0.05, **p < 0.01, ***p < 0.001, ****p < 0.0001. Experiments were repeated three times independently with similar results. Source numerical data are available in source data. Source data

Given the p16-dependent upregulation of PD-L1 in senescent cells, we wondered whether PD-L1 can serve as an extracellular marker to target senescent cells for depletion in vivo. To address this, we established a short-term LPS-mediated lung injury model, which resulted in the infiltration of p16 and PD-L1 double-positive immune cells in LPS-stimulated mice compared with the PBS control (Extended Data Fig. 4a–e). Specifically, AMs identified as CD11c^+^SiglecF^+^ cells were enriched for PD-L1 and p16 expression, concomitantly with our results in the models of chronic inflammation and ageing (Extended Data Fig. 4b,e). These experiments indicate that in response to stress, induction of endogenous p16 in vivo is associated with elevation of PD-L1 independently of the time scale of injury or identity of cell type responding to challenge. p16-mediated upregulation of PD-L1 in senescent cells makes it a potential target for monoclonal antibodies to stimulate anti-senescence immunity. Immunomodulatory PD-L1 antibodies can act either as antagonists to block the immune checkpoint PD1–PD-L1 axis or as agonists to enhance the immune response against target cells by engaging other immune system components^29^. Anti-PD-L1 antibodies engage activating Fcγ receptors on effector immune cells, which augments their in vivo activity (activating PD-L1 antibodies), while anti-PD1 antibodies block the axis in Fcγ-independent manner^30^. The treatment with activating anti-PD-L1 antibodies resulted in significant depletion of CD45^+^ cells positive for p16 and PD-L1, including a marked reduction of p16^+^PD-L1^+^ cells within the AM population, relative to the mice treated with isotype-matched control antibody (Extended Data Fig. 4f,g). Immune checkpoint blockade of PD1–PD-L1 axis can stimulate effector functions of lymphocytes, mainly CD8^+^ T cells. We tested if anti-PD-L1 antibody-mediated depletion of p16^+^PD-L1^+^ AMs had a functional consequence for T cell activity. We decided to narrow the analysis of T cells to bronchioalveolar fluid, the functional lung compartment of AM. The activating anti-PD-L1 antibody treatment significantly increased the percentage of CD8^+^ T cells showing expression of activation markers Inducible T cell COStimulator (ICOS) and PD1, known to be expressed during early T cell activation (Extended Data Fig. 4h). We have observed that anti-PD-L1 treatment also induced expression of IFNγ by CD8^+^ T cells marking the activation of their effector function (Extended Data Fig. 4i)^31^. Recently, it has been proposed that anti-PD1 improves senescence immunosurveillance in a CD8^+^ T cell-dependent manner^32^. Interestingly, our comparative study showed that only the treatment with agonist anti-PD-L1 antibody (and not antagonist anti-PD1 antibody) led to the depletion of p16^+^PD-L1^+^ cells in the inflamed lungs (Fig. 4e,f). This effect was accompanied by an increase of CD8^+^ T cells showing expression of activation markers ICOS and CD44 following anti-PD-L1 but not anti-PD1 treatment (Fig. 4g). These results suggest that in inflamed lungs, Fc-gamma receptor-positive cells (neutrophils, NK cells and monocytes) might be important players in the immune surveillance of senescent cells, either by direct cytotoxicity or by stimulating the activity of CD8^+^ T cells.

### Anti-PD-L1 antibody treatment depletes p16, PD-L1-positive senescent cells in ageing and chronic lung inflammation

**Fig. 5:**
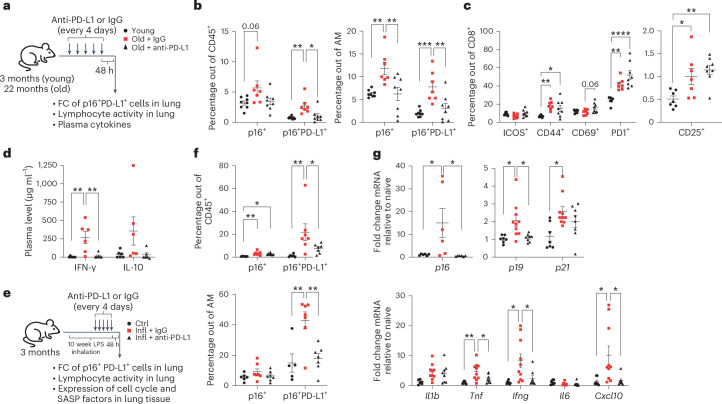
Anti-PD-L1 antibody depletes p16- and PD-L1-positive cells in ageing and chronic lung inflammation. **a**, Experimental setup included young or old mice that received an anti-PD-L1 or matched IgG control as indicated, and their lungs and blood were analysed 48 h after the last injection. FC, flow cytometry. **b**,**c**, Flow cytometry analysis of lungs from mice treated as in **a**, with a percentage of p16^+^PD-L1^+^ cells within CD45^+^ or AM (**b**) and a percentage of CD8^+^ T cells positive for ICOS, CD25, CD44, CD69 and PD1 (**c**). **d**, Plasma levels of IFN-γ and IL-10. **e**, Experimental setup included mice that were exposed three times a week for 10 weeks to LPS (Infl) inhalations and anti-PD-L1 or matched IgG control as indicated, and their lungs were analysed 48 h after the last inhalation. Naive mice were the control group (Ctrl). **f**, Percentage of p16^+^PD-L1^+^ cells within CD45^+^ or AM. **g**, Senescence-associated gene expression in the lungs of naive mice compared with the ones with chronic inflammation, treated with anti-PD-L1, or matched IgG control. For all experiments, statistical significance was calculated using one-way ANOVA; error bars, mean ± s.e.m. *P < 0.05, **P < 0.01, ***P < 0.001, ****P < 0.0001. In **a**–**d**, young (*n* = 7), old + IgG (*n* = 7) and old + anti-PD-L1 (*n* = 8) mice were used. In **e**–**g**, naive (*n* = 6–7), Infl + IgG (*n* = 7–10) and Infl + anti-PD-L1 (*n* = 7–8) mice samples were used. Experiments were repeated three times independently with similar results. Source numerical data are available in Source data. Source data

**Extended Data Fig. 5:**
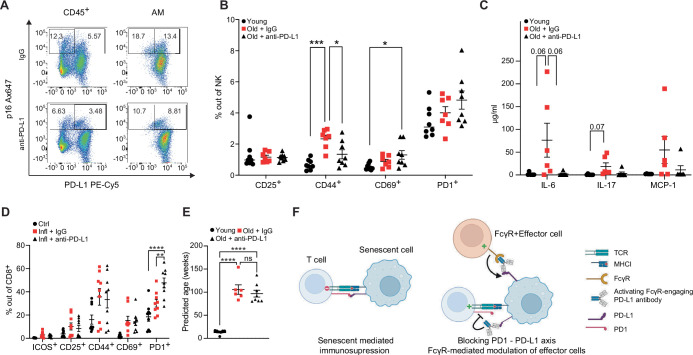
Anti-PD-L1 antibody treatment depletes p16, PD-L1^−^positive cells in aging and chronic lung inflammation. **(A-D)** Analysis of old mice treated with either anti-PD-L1 or isotype control (IgG). Young mice are the control group. **(**A-B) Flow cytometry analysis of the lungs. **(A)** Representative flow cytometry plots showing the gating strategy for p16^+^ PD-L1^+^ cells from immune cells (CD45^+^) and Alveolar Macrophage (AM) population. **(B)** Percentage of NK cells positive for CD25, CD44, CD69, and PD1. **(C)** Plasma levels of IL6, IL17, and MCP-1. **(D)** Predicted age based on the epigenetic clock of the peripheral immune system in the blood. **(E)** Flow cytometry analysis of mice with chronic lung inflammation (Infl) treated with either anti-PD-L1 or IgG control. Naive mice were the control group (Ctrl). Percentage of CD8 T cells positive for ICOS, CD25, CD44, CD69, and PD1. **(F)** Scheme of anti-PD-L1 antibody treatment of senescent cells and its effect. Statistical significance was calculated using one-way ANOVA; n = 6^−^9, Error bars, mean ± SEM. *p < 0.05, **p < 0.01, ***p < 0.001, ****p < 0.0001, (B-D) young (n = 7), old + IgG (n = 7), old + anti-PD-L1 (n = 8); (E-H) naive (n = 7-8), Infl + IgG (n = 7-10), Infl + anti-PD-L1 (n = 7-8). Experiments were repeated three times independently with similar results. Source numerical data are available in source data. Source data

Given that senescent cells promote inflammation, we tested whether anti-PD-L1 could mediate the elimination of senescent cells and attenuate inflammageing, senescence-promoted inflammation in ageing and chronic lung diseases. Activating anti-PD-L1 antibodies resulted in significant depletion of CD45^+^ positive for p16 and PD-L1 in aged lungs, including a marked reduction of p16^+^ and p16^+^PD-L1^+^ cells within the AM population, relative to the mice treated with isotype-matched control antibody (Fig. 5a,b and Extended Data Fig. 5a). It was accompanied by an increase of CD8^+^ T cells showing expression of activation markers CD25 and PD1, NK cells with marked upregulation of CD69, and decreased plasma levels of inflammatory cytokines (Fig. 5c,d and Extended Data Fig. 5b,c). Also, in chronic lung inflammation, we observed the anti-PD-L1-mediated depletion of p16^+^PD-L1^+^ cells within the general immune CD45^+^ and AM population (Fig. 5e,f), together with significant activation of CD8^+^ T cells expressing PD1 (Extended Data Fig. 5d), and decreased expression of cell cycle regulators and pro-inflammatory senescence-associated secretory phenotype factors (Fig. 5g, top and bottom, respectively). To understand if anti-PD-L1-mediated treatment could reverse age-dependent immunosenescence, we checked epigenetic ageing of the peripheral immune system in the blood by DNA methylation clock. There was no difference in the prediction of chronological age between aged mice treated with anti-PD-L1 antibodies and the isotype control (Extended Data Fig. 5e). Interestingly, recent reports suggest a relative stability of immune system dynamics in ageing over a short time^33^. In addition, anti-PD-L1-mediated depletion of senescent cells did not improve the capacity of the respiratory system upon chronic lung inflammation (data not shown). Collectively, our data suggests that anti-PD-L1 treatment improves immunosurveillance of senescent cells in physiological ageing and chronic lung inflammation and ameliorates senescence-associated pro-inflammation. However, the elimination of senescent cells by PD-L1 immune checkpoint blockade might not necessarily be a promising anti-ageing therapy, due to a limited effect on systemic age- and senescence-associated dysfunction in vivo.

## Discussion

We show that senescent cells upregulate the stability of PD-L1 in a p16-dependent manner and exploit the PD1/PD-L1 signalling axis as a general mechanism to escape immune surveillance. Accordingly, activating anti-PD-L1 antibodies enhances an immune response to deplete PD-L1-positive senescent cells in vivo (Extended Data Fig. 5f). Our results reveal the p16-dependent molecular mechanism that regulates PD-L1 stability in senescent cells. Specifically, p16-mediated inhibition of CDK4/6 downregulates ubiquitin-dependent proteasomal degradation of PD-L1, leading to its accumulation in senescent cells. p16-dependent upregulation of PD-L1 in senescent cells allows targeting of PD-L1 to improve immunosurveillance of senescent cells. Our findings reveal that the activating PD-L1 antibody engaging Fcγ receptors on effector cells reduces p16 and PD-L1 double-positive senescent cells in vivo and ameliorates senescence-associated inflammation.

Importantly, senescent cells are a heterogeneous population in vivo with differential expression of p16 and PD-L1, which might affect their PD-L1-mediated immunosurveillance. This is supported by our results showing that overexpression of p16 only moderately induces PD-L1 gene expression in mouse, but not human cells, which could be explained by enhanced accumulation of DNA damage in murine system in vitro (Fig. 3c and Extended Data Fig. 3b,c). PD-L1 expression is regulated by factors coupled with ageing, including DNA damage-mediated inflammation^24,32^. This explains why PD-L1 expression is enhanced in ageing, with p16 further promoting increased protein levels. Our study also underscores the important role of PD-L1 in the dynamics of senescent cell accumulation in ageing. Modelling of senescent cell turnover circuits indicated their saturation of removal in ageing^6^. Upregulation of PD-L1 stability in senescent cells can explain the intrinsic mechanism senescent cells employ to inhibit their removal and promote accumulation. Following damage, the buildup of PD-L1-positive senescent cells can be resolved efficiently in young compared with aged organisms as signals driving PD-L1 expression decline with injury resolution. Notably, PD-L1 is also upregulated on many cells regardless of p16 expression. Thus, while antibodies against PD-L1 are a promising strategy to eliminate PD-L1-positive senescent cells, they could also activate an immune response against PD-L1-positive non-senescent cells. Therefore, targeting PD-L1 in combination with other markers and pathways of senescent cells may offer therapeutic opportunities to treat senescence-mediated age-associated diseases.

p16-mediated elevation of PD-L1 in senescent cells could play a functional role in both pathological and physiological conditions. For instance, recently described p16-positive senescent cells in lung regeneration^34^ could upregulate PD-L1 to evade immune-mediated clearance, which otherwise would be detrimental to tissue integrity. However, persistent senescent cells with elevation of PD-L1 during chronic conditions might be associated with immunosuppression, possibly contributing to compromised immune responses and increased senescent cell burden in ageing and chronic inflammation.

The distribution of cells in the human body predicts macrophages to be one of the most abundant resident immune cells in non-lymphatic organs, comprising about 40% of the total immune cell population in the lungs^35^. Moreover, ageing of the immune system is characterized by decreased lymphopoiesis with reduced adaptive immunity and increased myelopoiesis, which can further contribute to an increase in macrophage population in some organs by several folds^36,37^. Interestingly, age-related skewing of the immune system towards myelopoiesis, which underlies inflammageing and myeloid-based pathologies, can be targeted by the depletion of myeloid-biased haematopoietic stem cells to rejuvenate aged immunity^38^. These findings highlight the central role of myeloid lineage, including macrophages, in the ageing of the immune system. Recently immunosenescence of myeloid cells, including senescent AM, was suggested to play an active role in systemic ageing and diseases^13,39–41^. Senescence of macrophages could contribute to their pro-inflammatory responses, but also compromised immune functions, leading to an increased senescence burden in tissues. Our data suggest that anti-PD-L1 antibodies could be a promising approach to activate the immune response, deplete senescent macrophages from tissues in ageing and chronic inflammation and ameliorate senescence-associated damage and systemic inflammation.

Blocking PD1 on T lymphocytes was shown to improve senescence surveillance in the liver^32^. Interestingly, our data show that only anti-PD-L1 antibodies engaging Fcγ receptors on effector cells, but not blocking anti-PD1 antibodies, led to the depletion of p16^+^PD-L1^+^ cells in the inflamed lungs. The PD1–PD-L1 checkpoint is a complex mechanism at the centre of the regulation of immune responses and, therefore, different antibodies targeting this axis may lead to non-symmetrical responses. The role of PD-L1 is not restricted to immunosuppression via its interaction with PD1 receptors on lymphocytes^42^. PD-L1 induces intracellular signalling that can enhance the survival of PD-L1-expressing cells, regulate their stress responses and confer resistance toward pro-apoptotic stimuli^43^. Our study highlights the complexity of the mechanism regulating immune surveillance of senescent cells and provides the basis for further studies.

In conclusion, we have identified that senescent cells upregulate the stability of PD-L1 in a p16-dependent manner and exploit the PD-L1 signalling to regulate immune responses. Senescent cells use immune checkpoint PD-L1 as an intrinsic mechanism to control their immunosurveillance. These results strongly suggest that persistent senescent cells expressing PD-L1 might lead to dysfunctional immune responses and an unbalanced inflammatory status, eventually resulting in chronic low-grade inflammation. Therefore, targeting PD-L1 on senescent cells not only enhances senescence immunosurveillance, but also alleviates senescence-associated inflammageing.

## Methods

### Cell culture

Mouse lung fibroblasts CCL-206, human lung fibroblasts IMR-90 and HEK293T were purchased from ATCC (CCL-206, CCL-186 and CRL-3216, respectively). The cells were cultured to 70% confluency in Dulbecco’s modified Eagle medium (DMEM), supplemented with 10% foetal bovine serum and 1% penicillin–streptomycin. To induce senescence, cells were treated with 50 μM etoposide (Sigma, E1383) for 48 h, washed three times with PBS and cultured for additional 5–7 days in DMEM. Replicative senescence (RIS) was induced by long-term passaging of the cells in tissue culture. The cells developed senescence phenotype after 35 population doublings. On the day of the experiment, the cells were detached using trypsin.

### Lentivirus production and infection

Generation of lentiviruses and their infection of cells was performed as described previously^44^. Lentiviruses were generated by co-transfecting HEK293T cells with 4 μg of pLX401-INK4A (AddGene, 121919) and 2 μg each of pLP/VSVG, pLP1 and pLP2 plasmids using Lipofectamine 2000 (Invitrogen, 11668019). Growth medium was exchanged the following day, and lentivirus-containing supernatant was collected 48 h later. CCL-206, IMR-90 and HEK293T cells were infected with the indicated viruses for 12 h, washed three times with PBS and cultured for an additional 24 h in RPMI medium. For selection, the cells were re-seeded in fresh RPMI medium with 1 μg ml^−1^ of puromycin (Gibco, A1113803) for 4–5 days. Doxycycline (Sigma-Aldrich, D3072) was added to the medium at a concentration of 5 or 10 μg ml^−1^ for inducible expression of the plasmid system.

### siRNA

Cells were transfected overnight with 50 nM of ON-TARGETplus SMARTpool small-interfering RNA (siRNA) targeting CDKN2A (L-011007-00-0005) or with non-targeting siRNA pool (D-001810-10-20) as a control (Dharmacon). At 24 h post-transfection, the remaining adherent cells were collected.

### CDK4/6 inhibitors

Abemaciclib (Pubchem, LY2835219) and palbociclib (Sigma-Aldrich, PZ0383) were dissolved in DMSO (vehicle) to yield 10 mM stock solutions and stored at 80 °C. IMR-90 cells were treated with DMEM supplemented with either 1uM abemaciclib, palbociclib or equivalent amount of DMSO for 48 h.

### Proteasome inhibition

IMR-90 cells were treated with DMEM supplemented with either 10 μM MG132 (Sigma-Aldrich, M7449) or equivalent amount of DMSO for 3 h.

### Immunoblot and immunoprecipitation assay

Cells were incubated in the radio-immunoprecipitation assay lysis buffer containing protease inhibitor cocktail (1:100) (Sigma-Aldrich, P8340) and phosphatase inhibitor cocktail (1:100) (Sigma, p5726) for 20 min on ice. Lysates were spun down for 15 min at 13,000 rpm and 4 °C, and protein concentrations were determined with bicinchoninic acid assay (Thermo Scientific). Equal amounts of protein were resolved by sodium dodecyl sulfate polyacrylamide gel electrophoresis and immunoblotted using β-TrCP (Cell Signalling Technology, CST-4394S), p16 (Abcam: Ab108349, human; Ab211542, mouse), vinculin (Abcam, Ab129002), PD-L1 (Cell Signalling Technology, CST-13684, human; Abcam, Ab213480, mouse) and appropriate HRP-conjugated secondary antibody, and with Europa Component Library visualization.

For immunoprecipitations analysis, cells were lysed in HNTG buffer (0.05 M HEPES pH 7.5, 10% glycerol, 0.15 M NaCl, 1% Triton X-100, 0.001 M ethylenediaminetetraacetic acid (EDTA), 0.001 M EGTA, 0.01 M NaF and 0.025 M β-glycerol phosphate) supplemented with protease inhibitor cocktail (1:100) (Sigma-Aldrich, P8340) and phosphatase inhibitor cocktail (1:100) (Sigma-Aldrich, P5726). Exaactly 2,000 μg of total cell lysates were incubated with previously coated protein A/G agarose beads (Santa Cruz Biotechnology, 2003) with anti-PD-L1 antibody (3 μg ml^−1^) (Cell Signalling Technology, 13684) overnight at 4 °C with gentle rotation. The beads were thoroughly washed with HNTG buffer and eluted with 6× sodium dodecyl sulfate loading buffer by boiling at 95 °C for 10 min. Ubiquitination of PD-L1 was measured by immunoblotting with anti-ubiquitin antibody (Santa Cruz Biotechnology, 8017).

### CHX chase assay

Twenty-four hours following transfection with siRNA, senescent IMR-90 cells were treated with 200 μM CHX (Sigma-Aldrich, C4859) for 3 h. Cells were lysed by incubation with 100 μl of radio-immunoprecipitation assay buffer (supplemented with PMSF and protease inhibitor cocktail) for 20 min on ice and protein concentrations were determined using bicinchoninic acid assay. A total of 10 μg of protein lysate was used to measure levels of PD-L1 by the enzyme-linked immunosorbent assay (ELISA), using the PD-L1/B7-H1 Quantikine ELISA Immunoassay kit (R&D, DB7H10), according to the manufacturer’s protocol. The optical density of each well was measured with the Infinite 200 plate reader (Tecan) at 450 nm with wavelength correction set at 540 nm. The experiment was performed twice and each sample was performed in duplicate.

### Immunofluorescent staining of human tissue microarray

Formalin-fixed, paraffin-embedded sections of human lung tissue microarray (US Biomax, LC487) were incubated at 60 °C for 60 min, deparaffinized and incubated in acetone for 7 min at −20 °C, followed by subsequent incubation with 3% H_2_O_2_ for 15 min at room temperature to block endogenous peroxidase activity. Antigen retrieval was performed in a microwave (3 min at full power, 1,000 W, then 20 min at 20% of full power) in Tris-EDTA buffer (pH 9.0). The slide was blocked with 20% NHS and 0.5% Triton in PBS and primary antibodies were diluted in 2% NHS and 0.5% Triton in PBS (p16, 1:30—Abcam, Ab108349; PD-L1, 1:100—Abcam, Ab213524) in a multiplexed manner with the Opal reagents (Akoya Bioscience), each one overnight at 4 °C. Following overnight incubation with the first primary antibody, the slide was washed with PBS, incubated in 2% NHS in PBS with secondary antibody conjugated to HRP (1:100) for 90 min, washed again and incubated with Opal reagents for 15 min. The slide was then washed and microwaved (as described above), washed, stained with the next primary antibody and with DAPI at the end of the cycle, and mounted. We used the following staining sequence: p16 → PD-L1 → DAPI. Each antibody was validated separately, and then multiplexed immunofluorescence was optimized to confirm that the antibody signal was not lost or changed due to the multistep protocol. Slides were imaged with an Eclipse Ni-U microscope (Nikon), connected to a colour camera (DS-Ri1, Nikon, ×20), and DAPI, Cy3 and Cy5 cubes. Images were analysed using the Fiji v2.6.0 software. The QuePath v0.4.4 software was used for the identification and quantification of cells positive for the fluorescent signal of each marker.

### Mice

Female C57BL/6 mice 10–14 weeks of age (young) or 24 months old (old) were used in all experiments. Mice were purchased from Harlan Laboratories. All mice were housed and maintained under specific pathogen-free conditions at the Weizmann Institute of Science in accordance with national animal care guidelines. The housing conditions were: 12-h dark/light cycle (lights on at 8:00), 22 °C temperature and 30–70% humidity. All procedures were performed in accordance with the protocols approved by the Weizmann Institute Animal Care and Use Committee (03320423-1, 06900820-2, 02720418-2, 05410621-3, 04000523-1 and 04040523-2).

### LPS exposure and treatment

For chronic LPS exposure, mice were exposed to an aerosolized PBS alone or PBS containing *Escherichia coli* LPS (0.5 mg ml^−1^; Sigma-Aldrich, L2630) for 30 min, three times a week for 10 weeks, in a custom-built cylindrical chamber as described previously^14^. For short-term 5-day LPS exposure, mice were exposed as in chronic exposure, but only for 5 constitutive days. Mice were killed and lungs were collected 48 h after the last exposure.

For the immune checkpoint blockade treatment mice received intravenous injection of 200 μg anti-PD-L1 (Ichorbio, ICH1086), 200 μg anti-PD1 (Ichorbio, ICH1091) or 200 μg isotype control IgG2b (Ichorbio, ICH2243).

In a short-term 5-day LPS exposure, mice were treated with immune checkpoint blockade on the second and fifth day of LPS inhalation. Old mice and mice undergoing chronic LPS exposure were treated with immune checkpoint blockade in five doses within 3 weeks, and the mice were euthanized 2 days after the final injection.

Bronchoalveolar lavage (BAL) fluid was collected from perfused lungs by double washing with 1 ml PBS through a tracheal catheter as previously described^14^.

### Measurement of cytokines levels in plasma

Blood was taken from the mice through cardiac puncture. To obtain plasma, blood samples were diluted 1:1 with PBS containing 1 mM EDTA upon the collection and then centrifuged at 3,400*g* for 15 min at 4 °C. Plasma levels of cytokines were measured by Milliplex MAP Mouse High Sensitivity T Cell Panel (cat no. MHSTCMAG-70K; Millipore) on Luminex (MAGPIX) following the manufacturer’s instructions. All samples were assayed in duplicate and mean values analysed. BELYSA v1.2 software (Millipore) was applied for data analysis. Concentrations are reported in pg ml^−1^.

### Epigenetic age predictions

Genomic DNA was isolated from whole blood using a Qiagen QIAamp DNA Mini and Blood Mini Kit (Qiagen) and DNA concentrations were measured with a NanoDrop 2000 spectrophotometer (Thermo Scientific). A total of 500 ng of genomic DNA was bisulfite converted with the Zymo Research Group EZ DNA Methylation Kit (Zymo Research). Pyrosequencing was performed with the PyroMark Q48 Autoprep system (Qiagen), and DNA methylation percentages were obtained for age-related CpGs in *Aspa*, *Wnt3a*, *Prima1* and *Hsf4* for the samples from young and old mice treated with anti-PD-L1 or matched isotype control. Primers, PCR conditions and targeted epigenetic age calculations were used, as described before^45^.

### Tissue dissociation

To achieve single-cell suspension from the lung, mice were euthanized by administration of xylazine/ketamine and then perfused by injecting cold PBS via the right ventricle before lung dissection. Lung tissues were dissected from mice, cut into small fragments and suspended in 1.5 ml of DMEM/F12 (Invitrogen, 11330-032) containing elastase (3 U ml^−1^, Worthington, LS002279), collagenase type IV (1 mg ml^−1^, Thermo Scientific, 17104019) and DNase I (0.5 mg ml^−1^, Roche, 10104159001) and incubated at 37 °C for 20 min with frequent agitation. After dissociation procedure, cells were washed with an equal volume of DMEM/F12 supplemented with 10% foetal bovine serum and 1% penicillin–streptomycin (Thermo Scientific), filtered through a 100-μm cell strainer and centrifuged at 380*g* for 5 min at 4 °C. Pelleted cells were resuspended in red blood cell ACK lysis buffer (Gibco, A1049201), incubated for 2 min at 25 °C, centrifuged at 380*g* for 5 min at 4 °C and then resuspended in ice-cold fluorescence-activated cell sorting (FACS) buffer (PBS supplemented with 2 mM EDTA, pH 8 and 0.5% bovine serum albumin).

### Flow cytometry

IMR-90 cells were stained with Zombie Aqua Viability fixable stain (423101) or Sytox Blue (Invitrogen, 34857) for evaluation of live/dead cells, followed by antibody Brilliant Violet 711-PD-L1 (329721) or isotype control (400353) staining (all from BioLegend).

Lung single-cell suspension was stained with anti-mouse CD16/32 (eBioscience, 14-0161-82) to block Fc receptors before labelling with fluorescent antibodies against cell-surface epitopes. For samples that were used for p16 intracellular staining, we used the following antibodies for extracellular staining: Brilliant Violet 605-CD45 (103140), FITC-CD11c (117306) and Brilliant Violet 421-SiglecF (155509) purchased from BioLegend. We used two clones of PD-L1 antibody (either Brilliant Violet 785-PD-L1, 124331, or PE-Cy5-PD-L1, 124344, with both cloning 10F.9G2, and PE-PD-L1, 153611, which clones MIH6) purchased from BioLegend, which yielded similar results. Then cells were fixed with 90% methanol for 10 min at 4 °C. All centrifugation steps after fixation were done at 850*g* for 5 min at 4 °C. For intracellular staining, cells were stained with p16 antibody (Abcam, Ab54210) conjugated to Alexa Fluor 647 fluorophore (Thermo Scientific, A20186). Cells were stained with Zombie Aqua Viability fixable stain for evaluation of live/dead cells. For characterization of immune subsets in BAL, we used the following antibodies: pacific blue-CD69 (104523), Brilliant Violet 605-ICOS (313537), Brilliant Violet 785-NK1.1 (108749), PerCP-CD19 (115531), FITC-CD3 (100204), PE-CD25 (102007), PE-Dazzle 595-TIGIT (142109), PE-Cy5-CD8 (100709), PE-Cy7-CTLA4 (106313), APC-LAG3 (125209), Spark Nir 685-CD4 (100475), Alexa Fluor 700-CD44 (103025), APC/Cy7-PD1 (135223) and APC Fire810-CD45 (103173). All antibodies were purchased from BioLegend and diluted 1:100 in FACS buffer before staining. Cell populations were recorded using LSR-II new (BD Biosciences) or Aurora (Cytec) and analysed using FlowJo v10 software (BD Biosciences) and Prism v7 software.

For imaging, flow cytometry cells were stained with FITC-CD45 (BioLegend, 103107), Brilliant Violet 786-PD-L1 (BioLegend, 124331) and Ax647-p16 (Abcam, Ab54210, conjugated to Alexa Fluor 647 fluorophore from Thermo Scientific, A20186). Before acquisition, cells were stained with DAPI and filtered through a 100 µm membrane. All antibodies were diluted 1:100 in FACS buffer before staining. Cells were acquired using ImageStreamX mark II (Amnis, part of EMD Milipore Merck) and image data analysis was performed using IDEAS v6.2 software as described in previously^6^.

### Mass cytometry

All antibodies used in the study, their corresponding clone, provider and catalogue number are listed in Supplementary Table 1. Antibodies were obtained in protein-free buffer. Custom metal-conjugated antibodies were generated using MaxPAR antibody labelling kits (Fluidigm) or the MIBItag Conjugation Kit (IONpath) according to the manufacturer’s instructions. After metal conjugation, the concentration of each antibody was determined with a Nanodrop (Thermo Scientific) and adjusted to 0.5 mg ml^−1^ with Antibody Stabilizer PBS (CANDOR Bioscience, 131050) for long-term storage at 4 °C. Lung single-cell suspension was washed once in 1 ml of cell staining buffer (CSB) (Fluidigm, 201068). To ensure homogeneous staining, 4 × 10^6^ cells from each sample were used. For viability staining, cells were incubated with 1.25 μM Cell-ID Cisplatin (Fluidigm, 201064) for 3 min before quenching with CSB. Before antibody staining, cells were incubated for 10 min at 4 °C with anti-mouse CD16/32 (Invitrogen, 14-0161-82) to block Fc receptors. Cells were stained with the epithelial or immune-centric antibodies for 45 min at 4 °C. An antibody cocktail of extracellular markers was prepared as a master mix and 50 μl of the cocktail was added to the samples resuspended in 50 μl of CSB. Cells were washed twice with CSB and permeabilized with fixation/permeabilization buffer (eBioscience, 88-8824-00). Then, samples were washed twice with CSB, incubated with 5% goat serum (Sigma-Aldrich, G-9023) and resuspended in 50 μl of CSB before the addition of 50 μl of cocktail of intracellular antibodies. For DNA-based detection, cells were stained with 125 nM Cell-ID Intercalator-Ir (Fluidigm, 201192 A) in PBS with 1.6% paraformaldehyde (Electron Microscopy Sciences, 15700) overnight at 4 °C. Cells were then washed once in CSB and twice in Maxpar Water (Fluidigm, 201069). For mass cytometry acquisition, samples were diluted to 3 × 10^5^ cells ml^−1^ in Maxpar Water containing 1:10 EQ Four Element Calibration Beads (Fluidigm, 201078) and filtered through a 35-μm filtermesh tube (Falcon). For acquisition CyTOF Helios system (Fluidigm) was used and samples were acquired at the rate of 200 events s^−1^. Data were collected as.fcs files. Data were normalized and concatented when necessary, via the CyTOF software v7.0 (Fluidigm). Then, the Cytobank platform (Beckman Coulter) was used to gate out the normalization beads according to the 140Ce channel. Next, several gates were applied to gate out live cells for further analysis. First, live single cells were gated using the cisplatin ^195^Pt, iridium DNA label in ^193^Ir, followed by the event length, and the Gaussian parameters of width, centre, offset and residual channels. To normalize data, a hyperbolic arcsine transformation (with a scale factor of five) was first applied. FlowSOM k-NN clustering and two-dimensional viSNE projections were calculated using Cytobank v9.0 software. Subsequently, mass cytometry data were analysed in Matematica (v14.0) and all custom-generated code is available in the AlonLabWIS git.

### Flow cytometry cell sorting

Cell populations were sorted using BD FACS Aria Fusion flow cytometer (BD Biosciences). Before sorting, all samples were filtered through a 70 mm nylon mesh. Populations that were sorted were epithelial cells (EpCam^+^CD31^−^CD45^−^Ter119^−^), AMs (CD45^+^CD11c^+^SiglecF^+^p16 high/low) and CD8a^+^ T cells (CD45^+^SiglecF^−^CD3^+^CD8a^+^). Sytox Blue (Invitrogen, 34857) or Aqua Zombie was used for viability staining. A range of 5,000–10,000 live cells were sorted into a low-bind eppendorf tube containing 50 µl of lysis/binding buffer (Invitrogen). Immediately after sorting, samples were spun down, snap frozen and stored at −80 °C until further processing.

To sort out lung epithelium, cells were stained with following antibodies: Brilliant Violet BV605-CD31 (102427), PE-CD45 (103106) and Alexa Fluor 488-EpCam (118210) all purchased from BioLegend and eFluor450-TER-119 (eBioscience, 48-5921-82). To sort out AMs, cells were stained with Brilliant Violet 605-CD45 (103140), FITC-CD11c (117306), Brilliant Violet 421-SiglecF (155509) and Brilliant Violet 786-PD-L1 (124331) all from BioLegend and p16 (Abcam, Ab54210) conjugated to Alexa Fluor 647 fluorophore (Thermo Scientific, A20186).

To sort out CD8a^+^ T cells, cells were stained with Brilliant Violet 605-CD45 (103140), Brilliant Violet 421-SiglecF (155509), FITC-CD3 (100204) and APC-CD8a (100711). All antibodies were purchased from BioLegend and diluted 1:100 in FACS buffer before staining.

### Preparation of libraries for RNA-seq

A total of 1 × 10^4^ cells of lung epithelium (EpCam^+^CD31^−^CD45^−^) were sorted into 50 μl of lysis/binding buffer (Life Technologies). mRNA was captured with 15 μl of Dynabeads oligo(dT) (Life Technologies), washed and eluted at 70 °C with 6.1 μl of 10 mM Tris-Cl (pH 7.5). Complementary DNA libraries were prepared from pooled samples of the same cell type (10,000 cells per sample) according to a bulk variation of massively parallel RNA single-cell sequencing (MARS-seq)^46^ and were sequenced on Illumina NextSeq 500 (Illumina). Intracellular staining and sequencing (INs-seq) libraries were prepared as previously described^27^, followed by bulk MARS-seq.

### RNA-seq analysis

Raw data were processed with the User-friendly Transcriptome Analysis Pipeline^47^. Only reads with unique mapping to the 3′ of RefSeq annotated genes (mm10, NCBI *Mus musculus* annotation release 109) were analysed. For differential gene expression analysis, we used DESeq2 (ref. ^48^), following standard workflow, to analyse RNA-seq count data derived from lung epithelial cells, comparing LPS to PBS. Genes with <30 unique molecular indentifiers across samples were pre-filtered. Differentially expressed genes were selected to have fold change > 1.25 and Benjamini–Hochberg-adjusted *P* < 0.05. For GSEA, we used DESeq2 (ref. ^38^) to derive gene fold changes for LPS versus PBS epithelial cells and for p16^+^ versus p16^−^ macrophages, controlling for treatment (LPS/PBS) as a covariant. We then applied GSEA to the ranked fold changes. We used Fast Gene Set Enrichment Analysis (‘fgsea’) library^49^ implemented in R to test for enrichment of gene sets (no. genes >10) from the mouse C5 v5p2 Gene Ontology collection of the Molecular Signature Database^50^.

### Real-time PCR analysis

mRNA was extracted from 5,000 CD8a^+^ T cells sorted into 50 µl of lysis/binding buffer (Invitrogen) and captured using Dynabeads oligo(dT) (Invitrogen) kit according to the manufacturer’s protocols. For lung tissue total RNA was extracted using Qiagen kit. For quantitative PCR analysis, mRNA was reverse transcribed using SuperScript II (Invitrogen, 11904018) and cDNA was diluted 1:10 for quantitative PCR analysis performed using the SYBR Green system. The relative gene expression was determined using the ΔΔCt method and normalization to *Actb*. We used four biological replicates for each condition. One-tailed *t*-tests were used to perform statistical analysis.

The following primers were used: mouse *Actb*—forward, 5′-GGAGGGGGTTGAGGTGTT-3′, reverse,

5′- TGTGCACTTTTATTGGTCTCAAG-3′; *Ifng*—forward,

5′- TGAACGCTACACACTGCATCTTGG-3′, reverse, 5′-CGACTCCTTTTCCGCTTCCTGAG-3′; *p16*—forward, 5′-TTGGGCGGGCACTGAATCTC-3′, reverse,

5′-AGTCTGTCTGCAGCGGACTC-3′; *p19*—forward, 5′-GCCGCACCGGAATCCT-3′; reverse,

5′- TTGAGCAGAAGAGCTGCTACGT-3′; *p21*—forward, 5′-GACAAGAGGCCCAGTACTTC-3′; reverse, 5′-GCTTGGAGTGATAGAAATCTGTC-3′; *Il-1b*—forward,

5′-GGAGAACCAAGCAACGACAAAATA-3′;reverse, 5′-TGGGGAACTCTGCAGACTCAAAC-3′;

*Tnf*—forward, 5′-CCACGCTCTTCTGTCTACTG-3′; reverse, 5′-GATGAGAGGGAGGCCATTTG-3′; *Il-6*—forward, 5′-TAGTCCTTCCTACCCCAATTTCC-3′; reverse,

5′-TTGGTCCTTAGCCACTCCTTC-3′; *CXCL10*—forward,

5′-CCATCAGCACCATGAACC-3′; reverse, 5′-TCCGGATTCAGACATCTC-3′; and *HPRT*—forward,

5′-TGACACTGGCAAAACAATGCA-3′; reverse, 5′-GGTCCTTTTCACCAGCAAGCT-3′.

### Statistics and reproducibility

For mice experiments, no statistical method was used to pre-determine sample sizes. In each experiment, the number of animals was chosen to have sufficient statistical power on the basis of the literature and experience^6,7,15^. For cell culture experiments, the sample size was determined to be at least *n* = 3 independent biological repeats, while in each experiment every sample had three technical repeats. Data are presented as means ± standard error of the mean (s.e.m.), unless otherwise noted. Comparisons between two groups were performed by an unpaired two-tailed Student’s *t*-test, unless otherwise noted. Comparisons between the three groups were performed by one-way analysis of variance (ANOVA). A chi-squared test was performed for RNA-seq analysis of differentially expressed genes. For consistency in comparisons, significance in all figures is denoted as follows: *P < 0.05, **P < 0.01, ***P < 0.001 and ****P < 0.0001.

### Reporting summary

Further information on research design is available in the Nature Portfolio Reporting Summary linked to this article.

## Online content

Any methods, additional references, Nature Portfolio reporting summaries, source data, extended data, supplementary information, acknowledgements, peer review information; details of author contributions and competing interests; and statements of data and code availability are available at 10.1038/s41556-024-01465-0.

### Supplementary information

Supplementary Information Gating strategy for flow cytometry analysis.

Reporting Summary

Supplementary Table 1 List of mass cytometry antibodies.

### Source data

Source Data Fig. 1 Statistical source data.

Source Data Fig. 2 Statistical source data.

Source Data Fig. 3 Statistical source data.

Source Data Fig. 3 Unprocessed western blots.

Source Data Fig. 4 Statistical source data.

Source Data Fig. 5 Statistical source data.

Source Data Extended Data Fig. 1 Statistical source data.

Source Data Extended Data Fig. 2 Statistical source data.

Source Data Extended Data Fig. 3 Statistical source data.

Source Data Extended Data Fig. 4 Statistical source data.

Source Data Extended Data Fig. 5 Statistical source data.

## Data Availability

All NGS sequencing data in this manuscript are available at NCBI GEO under the accession numbers GSE225285 (INseq data for alveolar macrophages) and GSE225286 (for lung epithelium). Mass cytometry data are available via juliamajewski GitHub at http://github.com/juliamajewski/p16-dependent-increase-of-PD-L1-stability-regulates-immunosurveillance-of-senescent-cells. All other data supporting the findings from this study are available from the corresponding authors on reasonable request. Source data are provided with this paper.

## Code availability

Custom-generated code for analysis of mass cytometry data is available via the AlonLabWIS GitHub at https://github.com/AlonLabWIS/Immune-checkpoint-keeps-senescent-cells-alive.git. Custom-generated code for analysis of RNA-seq data is available via the tomerlan GitHub at https://github.com/tomerlan/p16_PDL1.
