# Supplementary material for: p16-dependent increase of PD-L1 stability regulates immunosurveillance of senescent cells
Source: Nat Cell Biol. 2024 Aug 5;26(8):1336–45. doi: 10.1038/s41556-024-01465-0 (PMC11321988; doi:10.1038/s41556-024-01465-0)

nature cell biology

Supplementary information https://doi.org/10.1038/s41556-024-01465-0

p16-dependent increase of PD-L1 stability regulates immunosurveillance of senescent cells

In the format provided by the authors and unedited

1.Characterization of lymphocytes

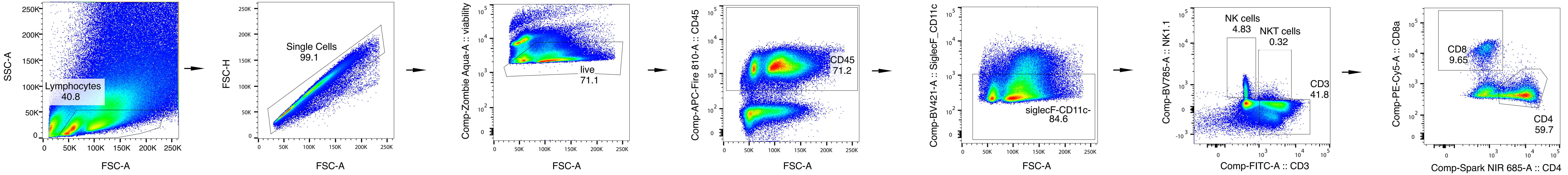

Gated on CD8 T cells, activation markers

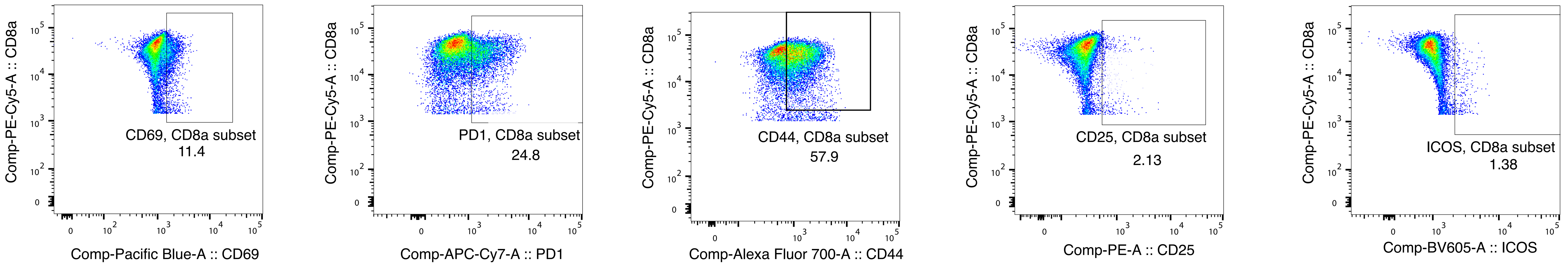

Gated on NK cells, activation markers

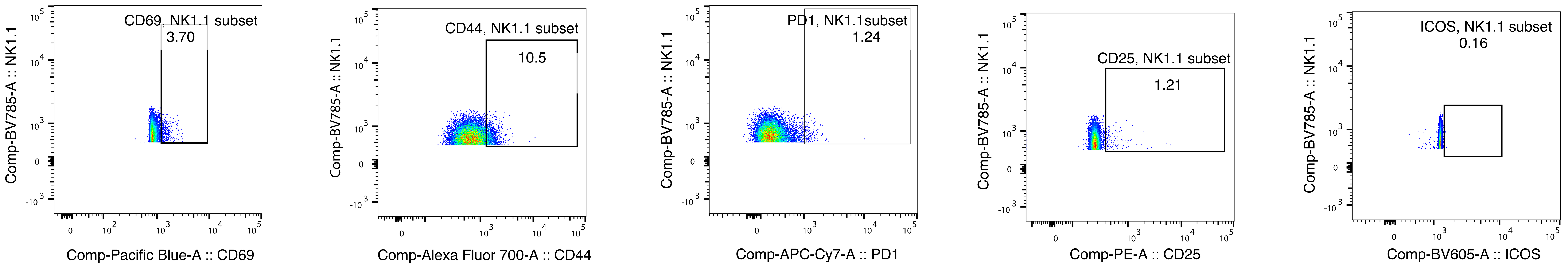

2. Identification of p16 and p16, PDL1-positive cells within CD45 and AM

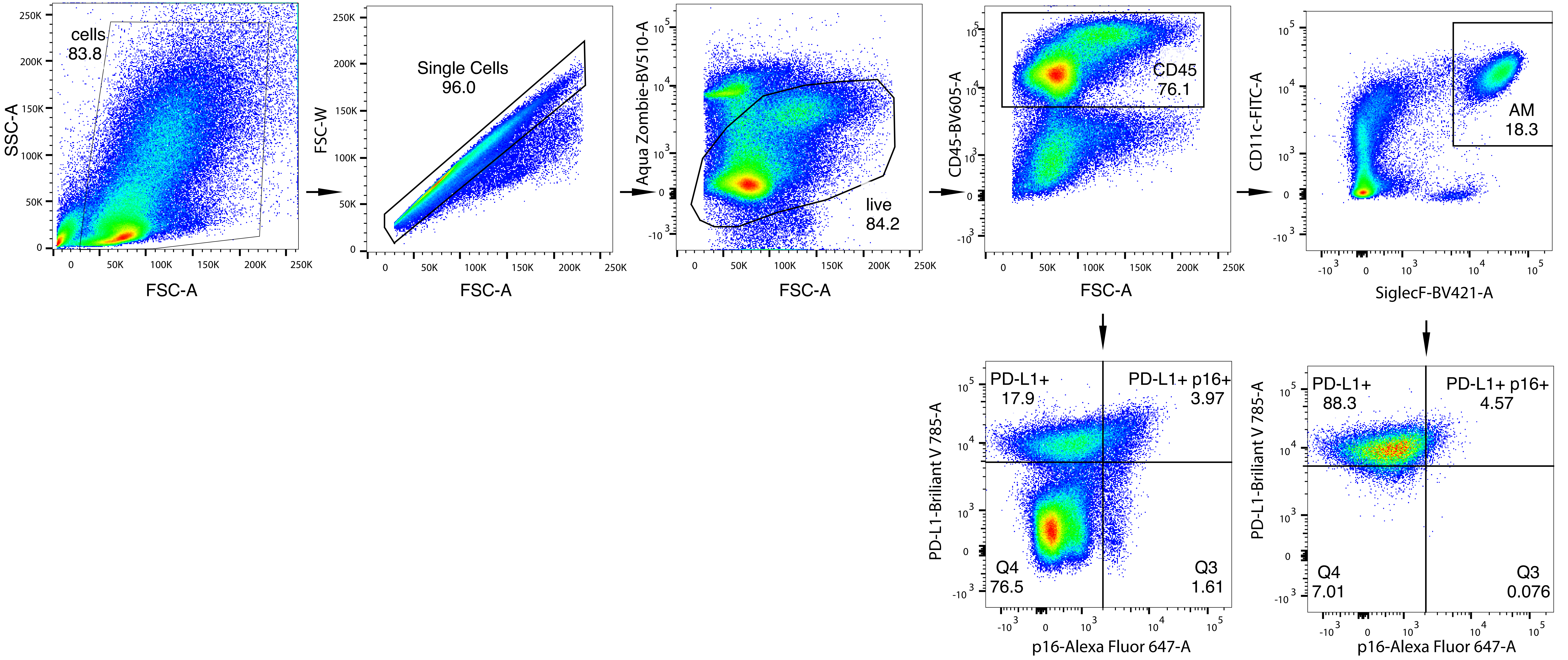

Supplement: Supplementary file 1 — Gating strategy for flow cytometry analysis. [file 41556_2024_1465_MOESM1_ESM.pdf]
