# Supplementary material for: p16-dependent increase of PD-L1 stability regulates immunosurveillance of senescent cells
Source: Nat Cell Biol. 2024 Aug 5;26(8):1336–45. doi: 10.1038/s41556-024-01465-0 (PMC11321988; doi:10.1038/s41556-024-01465-0)
Supplement: Supplementary file 2 — Reporting Summary [file 41556_2024_1465_MOESM2_ESM.pdf]

nature portfolio

Julia Majewska PhD
Corresponding author(s): Prof. Valery Krizhanovsky
Last updated by author(s): Dec 7, 2023

Reporting Summary

Nature Portfolio wishes to improve the reproducibility of the work that we publish. This form provides structure for consistency and transparency in reporting. For further information on Nature Portfolio policies, see our Editorial Policies and the Editorial Policy Checklist.

Statistics

For all statistical analyses, confirm that the following items are present in the figure legend, table legend, main text, or Methods section.

n/a	Confirmed	
☐	☒	The exact sample size (*n*) for each experimental group/condition, given as a discrete number and unit of measurement
☐	☒	A statement on whether measurements were taken from distinct samples or whether the same sample was measured repeatedly
☐	☒	The statistical test(s) used AND whether they are one- or two-sided<br>*Only common tests should be described solely by name; describe more complex techniques in the Methods section.*
☐	☒	A description of all covariates tested
☐	☒	A description of any assumptions or corrections, such as tests of normality and adjustment for multiple comparisons
☐	☒	A full description of the statistical parameters including central tendency (e.g. means) or other basic estimates (e.g. regression coefficient) AND variation (e.g. standard deviation) or associated estimates of uncertainty (e.g. confidence intervals)
☐	☒	For null hypothesis testing, the test statistic (e.g. *F*, *t*, *r*) with confidence intervals, effect sizes, degrees of freedom and *P* value noted<br>*Give P values as exact values whenever suitable.*
☒	☐	For Bayesian analysis, information on the choice of priors and Markov chain Monte Carlo settings
☒	☐	For hierarchical and complex designs, identification of the appropriate level for tests and full reporting of outcomes
☐	☒	Estimates of effect sizes (e.g. Cohen's *d*, Pearson's *r*), indicating how they were calculated

Our web collection on statistics for biologists contains articles on many of the points above.

Software and code

Policy information about availability of computer code

Data collection	For acquisition of mass cytometry data, CyTOF Helios II system (Fluidigm) was used.<br>For imaging flow cytometry, cells were acquired using ImageStreamX mark II (Amnis, Part of EMD Milipore Merck).<br>Flow cytometry data was recorded with LSR-II new (BD Biosciences) and Aurora (Cytec).<br>Cell sorting was done using BD FACSAria Fusion flow cytometer (BD Biosciences).<br>Immunofluorosent stainings were imaged with an Eclipse Ni-U microscope (Nikon), x20.<br>The optical density of quantikine ELISA Immunoassay was measured with the Infinite 200 plate reader (Tecan).<br>Serum levels of cytokines were measured on Luminex (MAGPIX).<br>Pyrosequencing was performed with the PyroMark Q48 Autoprep system (Qiagen).<br>cDNA libraries for RNAseq were sequenced on Illumina NextSeq500 (Illumina).
Data analysis	RNA seq - raw data was processed with the User-friendly Transcriptome Analysis Pipeline (UTAP), For differential gene expression analysis, we used DESeq2, For Gene set enrichment analysis, we used DESeq2 to derive gene fold-changes for LPS vs. PBS epithelial cells, and for p16+ vs. p16- macrophages, controlling for treatment (LPS/PBS) as a covariant. We then applied gene set enrichment analysis (GSEA) to the ranked fold-changes. We used Fast Gene Set Enrichment Analysis ("fgsea") library implemented in R software (v4.2.2) to test for enrichment of gene sets (#genes > 10) from the mouse C5 v5p2 gene ontology (GO) collection of the Molecular Signature Database.<br><br>For mass cytometry data was normalized, concatented when necessary, and pre-gated via the CyTOF software v7.0 (Fluidigm).<br>FlowSOM k-NN clustering and two-dimensions viSNE projections were calculated using Cytobank v9.0 software (Beckman Coulter).<br>Subsequenty mass cytometry data was analysed in Matematica (v14.0) and all custum generated code is available in the AlonLabWIS git (https://github.com/AlonLabWIS/Immune-checkpoint-keeps-senescent-cells-alive.git).

Custom generated code for analysis of RNA-Seq data is available at tomerlan git (https://github.com/tomerlan/p16_PDL1).

Flow cytometry data was analyzed using FlowJo v10 software (BD Biosciences) and Prism v7 software. Imaging flow cytometry data was analysed using IDEAS v6.2 software.
Cytokine levels were analysed by BELYSA v1.2 software (Millipore).
Immunofluorescence staining was analysed with Fiji v2.6.0 software and fluorescent signal was quantified by QuPath v0.4.4 software.

For manuscripts utilizing custom algorithms or software that are central to the research but not yet described in published literature, software must be made available to editors and reviewers. We strongly encourage code deposition in a community repository (e.g. GitHub). See the Nature Portfolio guidelines for submitting code & software for further information.

Data

Policy information about availability of data

All manuscripts must include a data availability statement. This statement should provide the following information, where applicable:

- Accession codes, unique identifiers, or web links for publicly available datasets
- A description of any restrictions on data availability
- For clinical datasets or third party data, please ensure that the statement adheres to our policy

All NGS sequencing data in this manuscript are available at NCBI GEO under the accession numbers GSE225285 (INseq data for alveolar macrophages), and GSE225286 (for lung epithelium). All mass cytometry data is available at juliamajewski GitHub (http://github.com/juliamajewski/p16-dependent-increase-of-PD-L1-stability-regulates-immunosurveillance-of-senescent-cells). All other data supporting the findings from this study are available from the corresponding author on reasonable request.
*We have not used any databases/dataset other then the ones generated in this study.

Human research participants

Policy information about studies involving human research participants and Sex and Gender in Research.

Reporting on sex and gender	Not applicable
Population characteristics	Not applicable
Recruitment	Not applicable
Ethics oversight	Not applicable

Note that full information on the approval of the study protocol must also be provided in the manuscript.

Field-specific reporting

Please select the one below that is the best fit for your research. If you are not sure, read the appropriate sections before making your selection.

☒ Life sciences ☐ Behavioural & social sciences ☐ Ecological, evolutionary & environmental sciences

For a reference copy of the document with all sections, see nature.com/documents/nr-reporting-summary-flat.pdf

Life sciences study design

All studies must disclose on these points even when the disclosure is negative.

Sample size	For mice experiemnts:<br>No statistical method was used to predetermine sample sizes, which were chosen to have sufficient statistical power based on the literature and past experience (Sagiv et al. Cell Reports 2018; Ovadya et al. Nat Commun 2019; Karin et al. Nat Comm 2019; Levi et al. Aging 2022).<br>For cell culture experiments, the sample size was determined to be at least n=3 independent biological repeats, while in each experiment every sample had 3 technical repeats.<br>Figure legends indicate the sample sizes and tests used to analyze each set of experiments.
Data exclusions	One aged and young mouse, could not be included in the CyTOF analysis due to plugs in Helios™ II CyTOF® that resulted in insufficient number of cells for analysis. Also one young mouse in ageing experiment with PD-L1 Treatment looks like technical outlier.
Replication	For chronic inflammation experiments, we conducted 4 (PBS, Control/Ctrl) and 4 (LPS, Infl) independent experiments, while in each experiment the number of mice in each group was at least n=3. The results were replicated in all the groups between all experiments. For aging experiments we had at least 4 mice in each group. For short term LPS inflammation experiments, we conducted at least 3 independent experiments with at least n=3 independent biological repeats.<br>For the cell culture experiments, we conducted at least n=3 independent biological repeats, while in each experiment every sample had 3 technical repeats.

Randomization	Animals were randomly assigned to experimental groups.
Blinding	During all experiments and analysis of results, investigators were blinded to the identity of the animals ,as well as to the identity of treatment (including cell culture experiments), when relevance.<br>Image analysis of human tissue microarray was performed by randomly choosing field of view.

Reporting for specific materials, systems and methods

We require information from authors about some types of materials, experimental systems and methods used in many studies. Here, indicate whether each material, system or method listed is relevant to your study. If you are not sure if a list item applies to your research, read the appropriate section before selecting a response.

Materials & experimental systems

n/a	Involved in the study
☐	☒ Antibodies
☐	☒ Eukaryotic cell lines
☒	☐ Palaeontology and archaeology
☐	☒ Animals and other organisms
☒	☐ Clinical data
☒	☐ Dual use research of concern

Methods

n/a	Involved in the study
☒	☐ ChIP-seq
☐	☒ Flow cytometry
☒	☐ MRI-based neuroimaging

Antibodies

Antibodies used	All fluorescent antibodies were diluted 1:100 with FACS buffer.<br>IMR-90 cells were stained with Zombie Aqua Viability fixable stain (#423101) for evaluation of live/dead cells, followed by antibody Brilliant Violet 711-PD-L1 (#329721) or isotype control (#400353) staining (all from Biolegend).<br><br>Lung single cell suspension was stained with anti-mouse CD16/32 (eBioscience, #14-0161-82) to block Fc receptors before labeling with fluorescent antibodies against cell-surface epitopes. For samples which were used for p16 intracellular staining, we used following antibodies for extracellular staining: Brilliant Violet 605-CD45 (#103140), FITC-CD11c (#117306), Brilliant Violet 421-SiglecF (#155509) purchased from Biolegend. We used two clones of PD-L1 antibody (Brilliant Violet 785-PD-L1, 10F.9G2, #124331, PE-PD-L1, MIH6, #153611) purchased from Biolegend, which yielded similar results. Then cells were fixed with 90% methanol for 10 min at 4°C. All centrifugation steps after fixation were done at 850g for 5 min at 4°C. For intracellular staining, cells were stained with p16 antibody (Abcam, #Ab54210) conjugated to Alexa Fluor 647 fluorophore (Thermo Scientific, #A20186). Cells were stained with Zombie Aqua Viability fixable stain for evaluation of live/dead cells. For characterization of immune subsets in BAL we used following antibodies: Pacific Blue-CD69 (#104523), Brilliant Violet 605-ICOS (#313537), Brilliant Violet 785-NK1.1 (#108749), PerCP-CD19 (#115531), FITC-CD3 (#100204), PE-CD25 (#102007), PE-Dazzle 595-TIGIT (#142109), PE-Cy5-CD8 (#100709), PE-Cy7-CTLA4 (#106313), APC-LAG3 (#125209), Spark Nir 685-CD4 (#100475), Alexa Fluor700-CD44 (#103025), APC/Cy7-PD1 (#135223), APC Fire810-CD45 (#103173) all from Biolegend.<br><br>For imaging flow-cytometry cells were stained with FITC-CD45 (Biolegend, #103107), Brilliant Violet 786-PD-L1 (Biolegend, #124331) and Ax647-p16 (Abcam, #Ab54210, conjugated to Alexa Fluor 647 fluorophore from Thermo Scientific, #A20186). Before acquisition, cells were stained with DAPI.<br><br>To sort out lung epithelium, cells were stained with following antibodies: Brilliant Violet BV605-CD31 (#102427), PE-CD45 (#103106), Alexa Fluor 488-EpCam (#118210) all purchased from BioLegend and eFluor450-TER-119 (eBioscience, #48-5921-82). To sort out alveolar macrophages, cells were stained with Brilliant Violet 605-CD45 (#103140), FITC-CD11c (#117306), Brilliant Violet 421-SiglecF (#155509), Brilliant Violet 786-PD-L1 (#124331) all from Biolegend and p16 (Abcam, #Ab54210) conjugated to Alexa Fluor 647 fluorophore (Thermo Scientific, #A20186). To sort out CD8a T cells, cells were stained with Brilliant Violet 605-CD45 (#103140), Brilliant Violet 421-SiglecF (#155509), FITC-CD3 (#100204), APC-CD8a (#100711) all from Biolegend.
Validation	In flow cytometry experiments, antibodies were used according to the manufacturer's instructions, and each antibody was compared individually to an unstained sample or fluorescence minus one control or/and isotype control.

Eukaryotic cell lines

Policy information about cell lines and Sex and Gender in Research

Cell line source(s)	Mouse CCL-206 fibroblasts, Human IMR-90 fibroblasts and HEK293T cells were obtained from the American Type Culture Collection (ATCC).
Authentication	Mouse CCL-206 fibroblasts, Human IMR-90 fibroblasts and HEK293T cells were authenticated by STR profiling conducted by the ATCC.
Mycoplasma contamination	All cell lines were tested for mycoplasma contamination.

Commonly misidentified lines (See ICLAC register) | We have not used any commonly misidentified cell lines.

Animals and other research organisms

Policy information about studies involving animals; ARRIVE guidelines recommended for reporting animal research, and Sex and Gender in Research

Laboratory animals

Female C57BL/6 mice 10 - 14 weeks of age (young) or 24 months old (old) were used in all experiments. All mice were housed and maintained under specific pathogen-free conditions at the Weizmann Institute of Science in accordance with national animal care guidelines. The housing conditions were: 12-hour dark/light cycle (lights on at 8 am), 22°C temperature, and 30-70% humidity.

For chronic LPS exposure, mice were exposed to an aerosolised PBS alone or PBS containing Escherichia coli LPS (0.5 mg/ml; Sigma, #L2630) for 30 min, 3 times a week for 10 weeks. For short-term 5-day LPS exposure, mice were exposed as in chronic exposure, but only for 5 constitutive days. Mice were sacrificed and lungs were harvested 48h after the last exposure.
For PD-L1 antibody treatment, mice received intravenous injection of 200 ug anti-PD-L1 (Ichorbio, #ICH1086), 200 ug of anti-PD1 (Ichorbio, #ICH1091) or 200 ug isotype control IgG2b (Ichorbio, #ICH2243) on the second and fifth day of short-term LPS inhalation. Old mice and mice undergoing chronic LPS exposure were treated with antibodies in 5 doses within 3 weeks, and the mice were euthanized two days after the final injection.

Wild animals

This study did not involve wild animals.

Reporting on sex

For this study female mice were chosen for the LPS experiments, as we have previously optimized Chronic Obstructive Pulmonary Disease model system in female mice following chronic exposure to LPS (Sagiv, 2018). To keep consistency, we also have used female mice for short term LPS experiments and ageing experiments.

Field-collected samples

This study did not involve samples collected from the field.

Ethics oversight

The Weizmann Institute of Science Animal Care and Use Committee (IACUC) approved all procedures described in this work.

Note that full information on the approval of the study protocol must also be provided in the manuscript.

Flow Cytometry

Plots

Confirm that:

☒ The axis labels state the marker and fluorochrome used (e.g. CD4-FITC).

☒ The axis scales are clearly visible. Include numbers along axes only for bottom left plot of group (a 'group' is an analysis of identical markers).

☒ All plots are contour plots with outliers or pseudocolor plots.

☒ A numerical value for number of cells or percentage (with statistics) is provided.

Methodology

Sample preparation

To achieve single cell suspension from the lung, mice were euthanized by administration of xylazine/ketamine and then perfused by injecting cold PBS via the right ventricle before lung dissection. Lung tissues were dissected from mice, cut into small fragments, and suspended in 1.5 ml of Dulbecco's modified Eagle medium/F12 medium (Invitrogen, #11330-032) containing elastase (3 U/ml, Worthington, #LS002279), collagenase type IV (1 mg/ml, Thermo Scientific, #17104019) and DNase I (0.5 mg/ml, Roche, #10104159001) and incubated at 37 °C for 20 min with frequent agitation. After dissociation procedure, cells were washed with an equal volume of DMEM/F12 supplemented with 10% FBS and 1% penicillin–streptomycin (Thermo Scientific), filtered through a 100-µm cell strainer, and centrifuged at 380g for 5 min at 4 °C. Pelleted cells were resuspended in red blood cell ACK lysis buffer (Gibco, #A1049201), incubated for 2 min at 25°C, centrifuged at 380g for 5 min at 4°C and then resuspended in ice-cold sorting buffer (PBS supplemented with 2mM ethylenediaminetetraacetic acid, pH 8 and 0.5% BSA).

Instrument

The cells were run in a LSR II Flow Cytometer (BD Biosciences) or Aurora (Cytec).
For imaging cell flow cytometry, cells were acquired using ImageStreamX mark II (Amnis, Part of EMD Milipore Merck) and analysis of the image data was performed using IDEAS 6.2 software.

Software

Data was collected in the BD FACSDiva™ software (BD Biosciences) and analyzed using the FlowJo v10 software (BD Biosciences) and Prism v7.

Cell population abundance

Samples were not sorted to post-sort fractions.

Gating strategy

Cells were identified by first gating to eliminate debris (FSC-A versus SSC-A), and then on singlets (FSC-H versus FSH-A). Live cells were gated based on their negative signal for DNA stain (either Sytox Blue or Aqua Zombie).

Immune cells were then identified by gating for CD45+ cells. The gating strategy for the immune subsets was performed as

follows: CD4 Tregs (CD45+/SiglecF-/CD3+/CD4+/Foxp3+), CD8 (CD45+/SiglecF-/CD3+/CD8+), NK cells (CD45+/SiglecF-CD3-/NK+) and alveolar macrophages (CD45+, CD11c+, Siglec-F+).
Epithelial cells (CD45-/CD31-/Epcam+).

Populations that were sorted were epithelial cells (EpCam+ CD31- CD45- Ter119-), alveolar macrophages (CD45+CD11c+SiglecF+ p16 high/low), and CD8a T cells (CD45+SiglecF-CD3+CD8a+). Sytox Blue (Invitrogen, #34857) or Aqua Zombie was used for viability staining.

☒ Tick this box to confirm that a figure exemplifying the gating strategy is provided in the Supplementary Information.
