# Supplementary material for: p16-dependent increase of PD-L1 stability regulates immunosurveillance of senescent cells
Source: Nat Cell Biol. 2024 Aug 5;26(8):1336–45. doi: 10.1038/s41556-024-01465-0 (PMC11321988; doi:10.1038/s41556-024-01465-0)

uncropped western blots

Fig. 3H

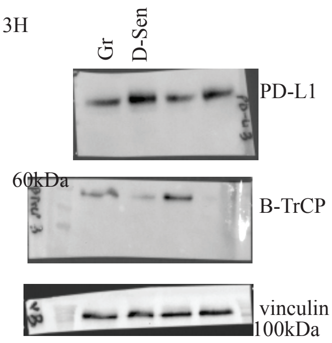

Fig. 3I

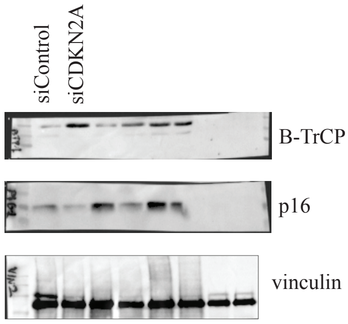

Fig. 3J

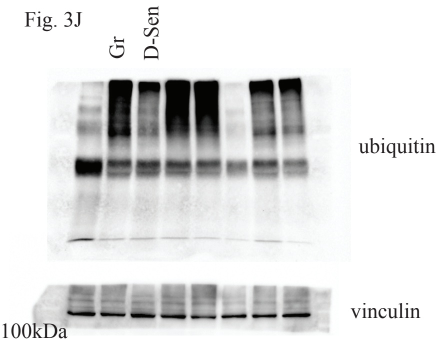

Fig. 3K

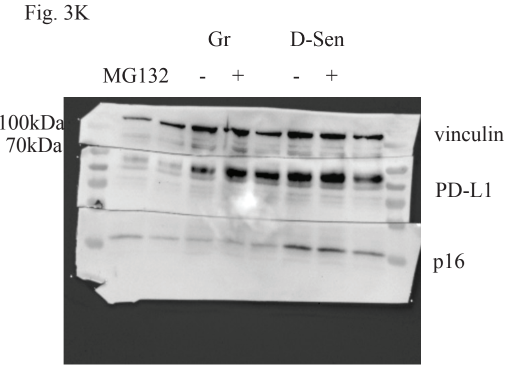

uncropped gels with red dotted line to border the area shown in respective figures

Fig. 3H

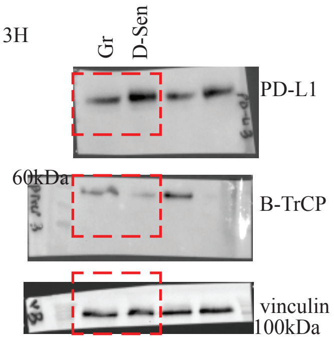

Fig. 3I

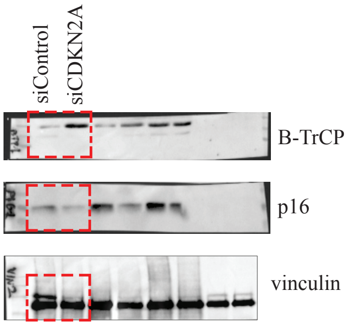

Fig. 3J

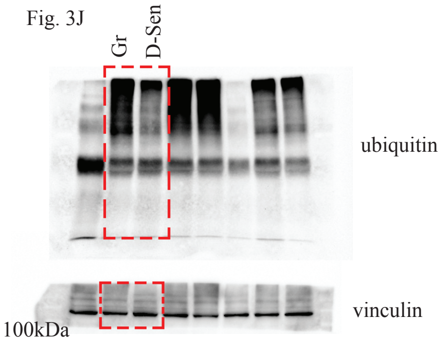

Fig. 3K

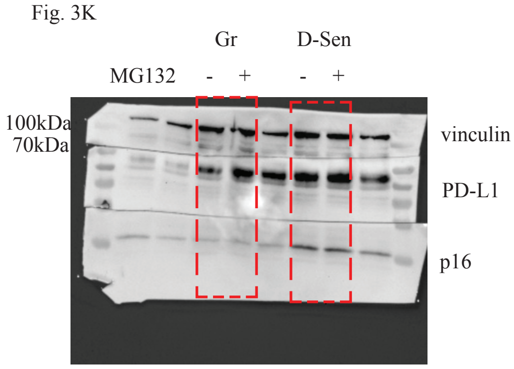

uncropped western blots

uncropped gels with red dotted line to border the area shown in respective figures

Extended Data Fig. 3B

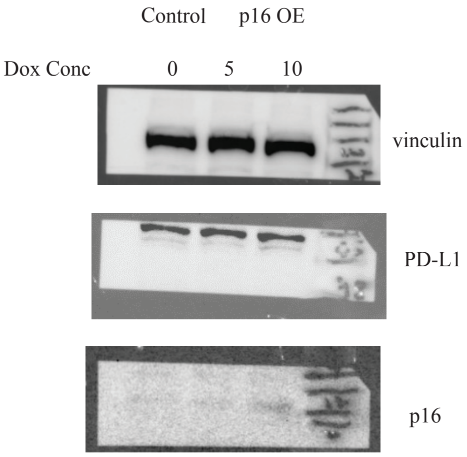

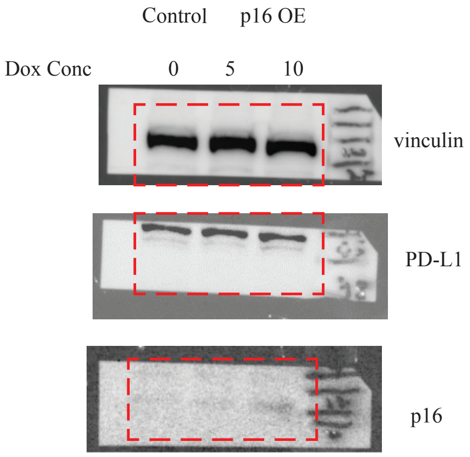

Extended Data Fig. 3C

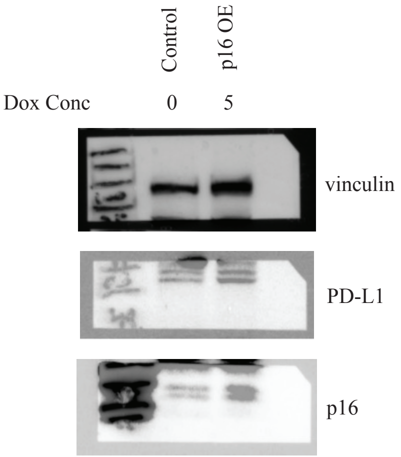

Extended Data Fig. 3C

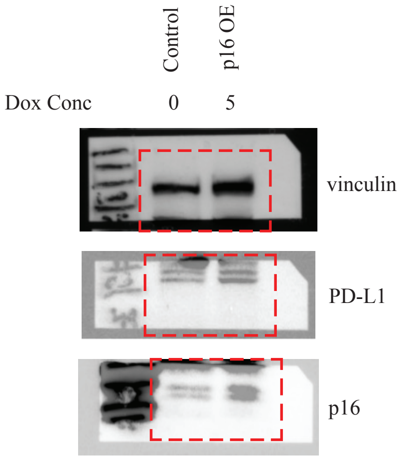

Supplement: Supplementary file 7 — Unprocessed western blots. [file 41556_2024_1465_MOESM7_ESM.pdf]
